# Inverse associations between dietary flavonoid and subclass intakes and frailty in U.S. adults

**DOI:** 10.3389/fnut.2025.1490998

**Published:** 2025-05-16

**Authors:** Shuangming Cai, Shan Huang, Huanshun Xiao, Yiping Luo

**Affiliations:** Department of MICU, Guangdong Women and Children Hospital, Guangzhou, China

**Keywords:** flavonoids, frailty, WQS, qgcomp, NHANES

## Abstract

**Objectives:**

Dietary flavonoids, known for their antioxidant and anti-inflammatory properties, may play a role in frailty prevention, but comprehensive population-based studies are lacking. This study aimed to examine the associations between dietary flavonoid intakes and the prevalence of frailty in a nationally representative sample of U.S. adults, and to identify the predominant flavonoid subclasses contributing to these associations.

**Methods:**

Cross-sectional data from 12,152 adults aged ≥20 years participating in the National Health and Nutrition Examination Survey (NHANES) 2007–2010 and 2017–2018 were analyzed. Dietary flavonoid intake was assessed using two 24-h dietary recalls. Frailty was defined using a 49-item frailty index. Weighted multivariate logistic regression models and restricted cubic spline analyses were employed to investigate the relationships between flavonoid intakes and frailty prevalence. Weighted quantile sum (WQS) regression and quantile g-computation (qgcomp) models were used to assess the mixed effects of flavonoid subclasses.

**Results:**

Higher intakes of total flavonoids (OR:0.79, 95% CI:0.65–0.95), anthocyanidins (OR:0.71, 95% CI:0.58–0.88), flavanones (OR:0.74, 95% CI:0.59–0.92), flavones (OR:0.76, 95% CI:0.59–0.97), and flavonols (OR:0.67, 95% CI:0.56–0.81) were significantly associated with lower prevalence of frailty after adjusting for confounders. Non-linear inverse associations were observed for total flavonoids and flavonols. The WQS model revealed that the mixture of flavonoid subclasses was inversely associated with frailty odds (OR: 0.58, 95% CI: 0.48–0.71, *p* < 0.001), with flavones, flavonols, and anthocyanidins as the top contributors. The qgcomp model confirmed these findings but highlighted potential opposing effects among subclasses.

**Conclusion:**

This comprehensive analysis provides evidence that higher dietary flavonoid intakes, particularly flavones, flavonols, and anthocyanidins, are associated with lower prevalence of frailty in U.S. adults. These findings suggest that flavonoid-rich diets may be a promising strategy for frailty prevention, warranting further investigation through prospective cohort studies and randomized controlled trials.

## 1 Introduction

The world is experiencing an unprecedented demographic shift toward an aging population. By 2050, the number of individuals aged 60 years and older is expected to double, reaching approximately 2.1 billion, with the most rapid growth occurring in low- and middle-income countries (1). This rapid aging of the global population presents significant challenges to healthcare systems, social structures, and economies worldwide. One of the most pressing concerns associated with this demographic transition is the increased prevalence of age-related conditions, with frailty emerging as a key issue (2).

Frailty is a clinical syndrome characterized by decreased physiological reserve and increased vulnerability to stressors. Frailty develops through multiple interconnected mechanisms: physiologically via inflammation, oxidative stress, and mitochondrial dysfunction (3); biomechanically through reduced muscle strength and altered mobility (4); and physically via decreased activity (5). These processes, marked by elevated inflammatory cytokines, oxidative stress, and impaired antioxidant defenses, represent potential targets for nutritional interventions to mitigate age-related decline. It is typically defined by the presence of three or more of the following criteria: unintentional weight loss, exhaustion, weakness, slow walking speed, and low physical activity (6). This syndrome is associated not only with an increased risk of adverse health outcomes, including falls, hospitalization, disability, and mortality (7), but also with greater utilization of healthcare resources. The incidence of frailty varies across different regions and populations (8, 9), and also fluctuates with age. In the UK Biobank study, which included 493,737 participants aged 37–73 years, 16,538 (3%) were considered frail, while 185,360 (38%) were classified as pre-frail. The prevalence of frailty and pre-frailty increased with age in both women and men. For women, it rose from 41% in the 37–45 year age group to 47% in the 65–73 year age group. For men, it increased from 37% in the 37–45 year age group to 43% in the 65–73 year age group (10). In the United States, a study of middle-aged adults from Baltimore found that frailty is present even in younger populations, with prevalence rates of 7.2% in 35–44 year-olds, 10.0% in 45–55 year-olds, and 15.4% in 55–64 year-olds, underscoring that frailty is not exclusively a geriatric syndrome but can emerge in mid-life. This study also revealed that poverty more than doubled the odds of frailty (OR = 2.34) and that frailty was independently associated with mortality (HR = 2.30) beyond the influence of demographic factors (11). Additionally, data from the Health and Retirement Study demonstrated that frailty incidence in US adults aged ≥50 years showed a fluctuating but decreasing trend from 2004–2020, with a peak occurrence following the Great Recession (2007–2009), suggesting economic crises may impact frailty rates. Nearly 20% of frailty incidence was attributed to modifiable lifestyle factors including smoking, physical inactivity, and sleep problems (12). Despite its prevalence and impact, effective treatments for frailty are lacking, underscoring the importance of research in frailty prevention and treatment.

Current interventions for frailty, including physical exercise (13), nutritional support (14), and pharmacological treatments (15), have shown limited success due to various challenges. These include issues with long-term adherence, potential side effects of medications, and the need for sustained, multidisciplinary approaches (16). Given these limitations, there is growing interest in exploring more feasible, acceptable, and cost-effective approaches to managing frailty. Dietary modifications, particularly those focusing on bioactive compounds with potential health benefits, have emerged as a promising avenue for intervention (17). Recent studies have highlighted the potential of plant-derived bioactives in enhancing therapeutic outcomes and preventing age-related conditions. For instance, research on *Polygonum minus* has demonstrated its synergistic effects with conventional chemotherapeutic agents like 5-fluorouracil, underscoring the therapeutic potential of combining natural compounds with medical interventions (18). Similarly, dietary bioactives such as flavonoids have been shown to enhance the efficacy of treatments and improve patient outcomes (19). These findings suggest that dietary modifications, particularly those emphasizing bioactive compounds, may offer promising strategies for promoting health and resilience in aging populations.

Flavonoids, a diverse group of naturally occurring polyphenolic compounds abundantly found in fruits, vegetables, tea, and other plant-based foods, have emerged as potential dietary factors that may influence frailty risk (20). These compounds are renowned for their potent antioxidant, anti-inflammatory, and anti-apoptotic properties, which have been associated with a range of health benefits (21–24). Extensive research has linked flavonoid consumption to reduced risks of chronic diseases, including cardiovascular disorders (25), certain cancers (26), and neurodegenerative conditions (27). Different flavonoid subclasses exhibit distinct biological mechanisms that may influence frailty development. For instance, flavonols, particularly quercetin, have demonstrated strong antioxidant properties and the ability to modulate inflammatory pathways through NF-κB inhibition (28, 29). Anthocyanidins have shown particular efficacy in improving mitochondrial function and reducing oxidative stress (30–32). These mechanistic differences may explain the varying associations observed between different flavonoid subclasses and health outcomes. These studies suggest that the consumption of a diverse array of flavonoid-rich foods may bolster overall health and potentially mitigate the risk of age-related decline.

A limited body of existing research demonstrates a beneficial relationship between total flavonoid intake or specific flavonoid subclasses and frailty. For instance, Munguia et al. observed that regular flavonoid consumption positively affected blood oxidative stress and inflammation endpoints, cardiometabolic risk markers, physical performance, and quality of life in elderly individuals, potentially mitigating frailty development (33). A recent hypothesis-generating study by Oei et al. found that while total flavonoid intake was not associated with frailty onset in adults, higher intake of flavonols, particularly quercetin, was associated with lower odds of frailty onset (34). These findings underscore the importance of assessing specific subclasses of flavonoids and highlight the potential of dietary flavonols as a strategy to prevent the development of frailty.

Despite these promising findings, there remains a paucity of comprehensive population-based studies examining the association between dietary flavonoid intake and the prevalence of frailty. Current research literature has several limitations, including a limited understanding of flavonoid subclass-specific effects, insufficient evidence from large, representative populations, and a lack of comprehensive mixture analyses. To address these knowledge gaps and provide more conclusive evidence, large-scale population studies utilizing comprehensive dietary flavonoid data are critically needed. Such research would not only elucidate the relationship between flavonoid intake and frailty but also potentially inform dietary recommendations for frailty prevention and management.

Based on the aforementioned background, the present study aims to examine the associations between dietary flavonoid intake, including specific flavonoid subclasses, and the prevalence of frailty in U.S. adults. Our study addresses these research gaps through detailed subclass analysis, utilizing nationally representative data from the National Health and Nutrition Examination Survey (NHANES) spanning the years 2007–2010 and 2017–2018, and employing innovative statistical approaches to examine mixture effects. The NHANES database was selected for this study due to its nationally representative sampling, standardized dietary assessment methods, and comprehensive health examination data. The specific cycles (2007–2008, 2009–2010, and 2017–2018) were chosen because they represent the most recent periods with complete flavonoid intake data and consistent dietary assessment methodologies. These cycles also provide adequate sample size for robust statistical analysis while minimizing potential temporal changes in dietary patterns.

## 2 Materials and methods

### 2.1 Study population

**Figure 1:**
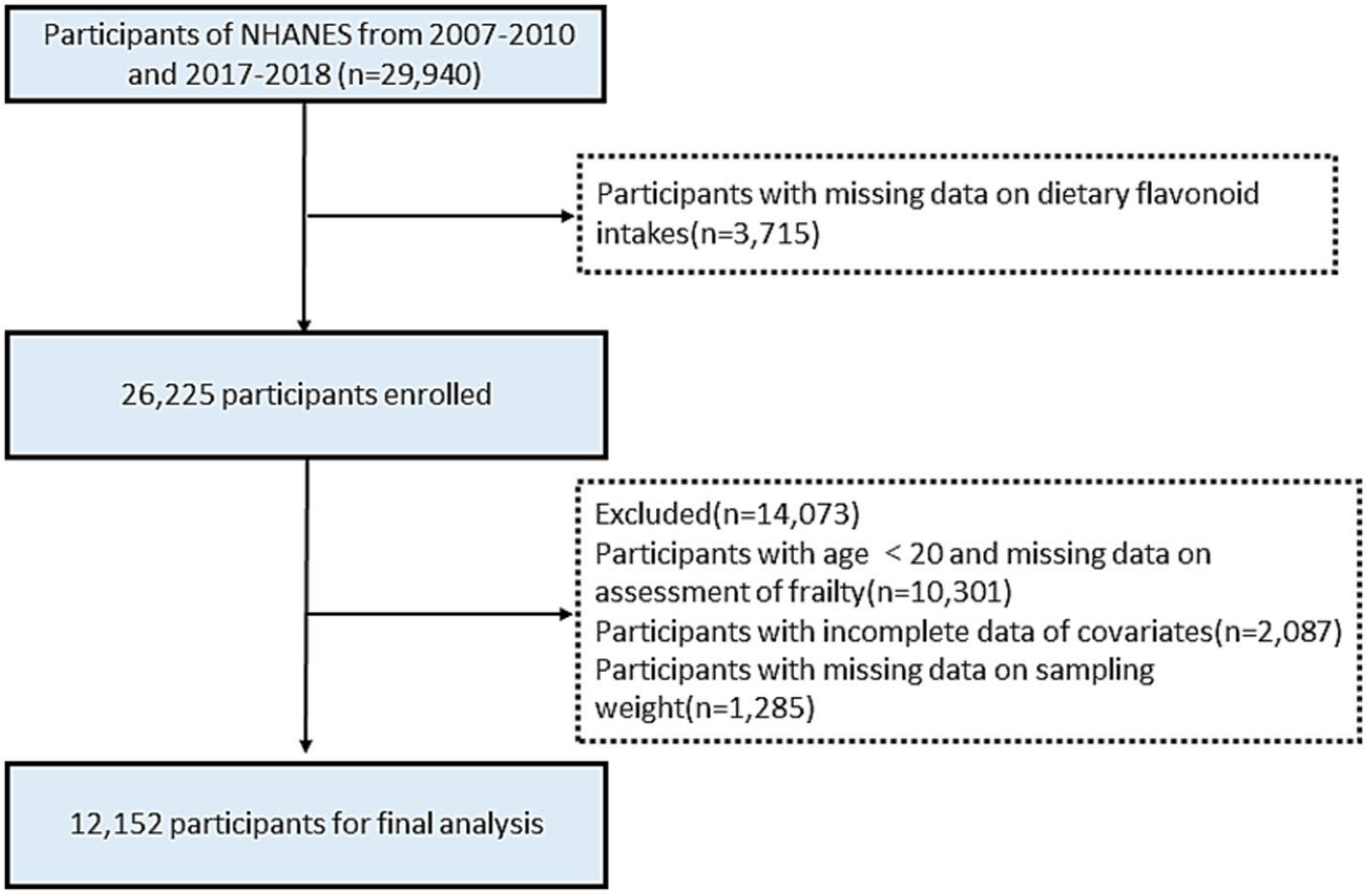
Flowchart of the study participants.

The NHANES database is an annual cross-sectional survey conducted annually in the United States, comprising a health interview survey and a physical health survey of participants. All participants provided written informed consent and study procedures were approved by the National Center for Health Statistics Research Ethics Review Board (Protocol Number: Protocol #2005–06 and Protocol #2011–17). For this study, we used publicly available data for three NHANES cycles (2007–2008, 2009–2010, 2017–2018), in which 29,940 people were surveyed over the three periods. Participants with missing data on flavonoid intake were excluded (*n* = 3,715). Participants with age <20 and missing data on frailty assessment were excluded (*n* = 10,301). Participants with incomplete data of covariates were excluded (*n* = 2,087). Finally, after removing individuals with missing data on sampling weight (*n* = 1,285), a total of 12,152 participants were included in the analysis (Figure 1). As a cross-sectional study, our findings cannot establish causal relationships between flavonoid intake and frailty. This limitation is inherent to the study design and highlights the need for prospective cohort studies and randomized controlled trials to confirm these associations.

### 2.2 Assessment of dietary flavonoid intakes

Dietary flavonoid intake data were obtained from the United States Department of Agriculture (USDA) Food and Nutrient Database for Dietary Studies (FNDDS).^1^ This database represents the most comprehensive and standardized database for U.S. dietary studies, which provides information on the flavonoid content data of over 7,000 foods and beverages, and was developed to process dietary data collected from What We Eat in America (WWEIA), a component of the National Health and Nutrition Examination Survey (NHANES) (35). A total of 7,273 food codes were used to extract values for total flavonoids and six flavonoid subclasses. Data on flavonoid intake for the years 2007–2010 and 2017–2018 were gathered for our study.

The assessment focused on six major flavonoid subclasses: isoflavones, anthocyanidins, flavan-3-ols, flavanones, flavones, and flavonols. These subclasses encompass 29 individual flavonoids, including compounds such as daidzein, cyanidin, epicatechin, hesperetin, apigenin, and quercetin. We acknowledge that 24-h dietary recalls may introduce misclassification bias due to day-to-day variations in dietary intake and potential recall errors. To minimize this limitation, we used the mean value of dietary flavonoid intake from two 24-h recalls as the final dietary flavonoid intake and included total energy intake as a covariate in our analyses. For this study, total flavonoids intake was calculated as the sum of the mean values from these six subclasses (36).

### 2.3 Assessment of frailty

Frailty was the primary outcome, assessed using the frailty index established by Wael Sabbah et al. (37), which incorporates 49 diagnostic criteria following Searle and colleagues’ standard procedures (38). The 49-item frailty index was chosen over other assessments (e.g., Fried’s phenotype) because it provides a more comprehensive evaluation of frailty, incorporating multiple physiological systems and both objective and subjective measures. This approach aligns with the current understanding of frailty as a multidimensional syndrome. These diagnostic items cover cognition function, dependency, depression, comorbidities, medical status, hospital usage, general health, anthropometrics, and laboratory tests. Each entry has adjudication criteria, assigning a score between 0 (no defect present) and 1 (most severe defect). The frailty index is calculated by dividing the total score by the number of items answered. To ensure the quality of frailty diagnoses, we included participants who responded to at least 80% of the items. The frailty index greater than or equal to 0.25 is defined as frailty (39). Detailed the scoring criteria are provided in the Supplementary Table S1. We acknowledge that self-reported components may introduce reporting bias; however, the index’s inclusion of objective measurements (laboratory tests, physical measurements) helps balance this limitation. Furthermore, this index has been validated in previous NHANES analyses and shows strong correlations with adverse health outcomes (37, 40).

### 2.4 Covariates

Numerous covariates were considered in our analysis. These variables, collected through home interviews or laboratory measurements, encompassed age, sex, race/ethnicity, smoking status, drinking status, body mass index (BMI), waist circumference (WC), estimated glomerular filtration rate (eGFR), energy intake levels, supplement use, and several chronic diseases. Age were categorized into three groups: <40 years, 40–59 years, and>59 years. Race was categorized into five groups: Non-Hispanic White, Mexican American, Non-Hispanic Black, Other Hispanic, Other Race. Smoking status was classified as never smoker, former smoker, and current smoker. Drinking status was classified as non-drinker, former drinker, and current drinker. The BMI was divided into 3 groups: <25, 25–30, and > 30 kg/m^2^. The estimated glomerular filtration rate (eGFR) was calculated using the creatinine equation of the Chronic Kidney Disease Epidemiology Collaboration (41). Total energy intakes were divided into quantiles (Q1, Q2, and Q3). We obtained information on supplement use using self-reported questionnaires. Chronic diseases, such as stroke, diabetes mellitus (DM), hyperlipidemia, hypertension, and depression were also defined.

### 2.5 Statistical analysis

According to the NHANES analytic guidelines, primary sampling units (SDMVPSU), stratification (SDMVSTRA), and sampling weight (WTDR2D, dietary two-day sample weight) were incorporated to generate nationally representative estimates, and the individuals’ original dietary two-day sample weight divided by three was adopted as the final weight since three survey cycles were combined in this study. Continuous variables are presented as weighted means (SE), and categorical variables are reported as number and weighted proportions. In our study, participants with missing data on key variables were excluded from the analysis. No multiple imputation or other methods were used.

In order to effectively control confounding factors, stepped logistic regression models were constructed to obtain the multivariable-adjusted effects of dietary flavonoid intakes on the odds of frailty. The stepwise model adjustments were constructed based on theoretical frameworks and previous literature. Model 1 was the original model without adjustment; Model 2 adjusted for age, sex and ethnicity; Model 3 adjusted for all the factors in Model 2 and further adjusted for smoking status, drinking status, BMI, total energy intakes, and supplement use. Trend tests (p for trend) were performed by entering the dietary flavonoid intake (quartile-categorical) as a continuous variable and rerunning the corresponding regression models. The restricted cubic spline (RCS) models with three knots (5th, 50th, and 95th percentiles) were additionally utilized to examine their dose–response relationships. The selection of these three knot positions was based on simulation studies that demonstrated optimal performance in detecting non-linear relationships while minimizing the risk of overfitting.

The weighted quantile sum (WQS) regression model was utilized to estimate the mixed effects of six flavonoid subclasses and identify the predominant types through the calculated WQS index (42). This index was comprised of weighted sums of single flavonoid concentrations and reflected the mixture exposure level. The weight calculation for each flavonoid intake involved bootstrap sampling (*n* = 2000), dividing the data into training (40% samples) and test (60% samples) sets. The training set determined the weights, while the test set calculated mixture significance. The final result ranged from 0 to 1 and was interpreted as the simultaneous effect of a one-quantile increase of the mixture on frailty. Since the WQS model assumes that all exposures must have the same direction of effect with the outcome, the quantile g-computation (qgcomp) model, a generalization and extension to WQS, was further adopted as a sensitivity analysis (43). This approach addresses the challenges of analyzing complex exposure mixtures through quantile-based standardization of exposure variables while assuming monotonic exposure-response relationships. Unlike traditional regression methods, it effectively handles correlated exposures and reduces dimensionality while maintaining result interpretability. A key feature of qgcomp is its ability to accommodate both positive and negative weights, where these weights represent the proportion of negative or positive partial effects due to specific exposures, with the sums of negative and positive weights each normalized to 1. This flexibility allows for a more nuanced interpretation of mixture effects, particularly when directional homogeneity does not hold across all exposure components. We selected WQS and qgcomp models because they are specifically designed to assess the effects of exposure mixtures while accounting for potential interactions and cumulative effects. Unlike principal component analysis, which reduces dimensionality without preserving interpretability, WQS and qgcomp provide interpretable weights for each component in the mixture, allowing us to identify the relative contributions of individual flavonoid subclasses.

All analyses were performed using R (version 4.3.3) and *p*-values less than 0.05 were considered statistically significant.

## 3 Results

### 3.1 The characteristics of study participants

The study included up to 12,152 adults from three 2-year cycles of NHANES for the association analysis of dietary flavonoids with frailty. Of the study participants, 26.45% were over 59 years of age, 47.58% were male, 67.89% were non-Hispanic White. The demographic characteristics of the study participants are presented in Table 1, divided into non-frailty (including 10,441 participants) and frailty (including 1,711 participants) groups. Frail individuals were more likely to be those with advanced age, female, non-Hispanic White, lower income, non-smokers, current drinkers, hyperlipidemia, and hypertension patients, compared to those non-frail participants (all *p* < 0.01).

**Table 1:** Survey-weighted, sociodemographic and health status characteristics of study participants.

Characteristics	Total (*n* = 12,152)	Non-frailty (*n* = 10,441)	Frailty (*n* = 1,711)	*p* value
Age, years	46.90 (0.33)	45.72 (0.35)	57.19 (0.55)	<0.0001
Age group, %				<0.0001
<40	3,679 (35.49)	3,514 (38.17)	165 (12.10)	
40–59	4,088 (38.05)	3,491 (37.66)	597 (41.44)	
>59	4,385 (26.45)	3,436 (24.16)	949 (46.46)	
Sex, %				<0.0001
Female	6,310 (52.42)	5,287 (51.22)	1,023 (62.84)	
Male	5,842 (47.58)	5,154 (48.78)	688 (37.16)	
Race/ethnicity, %				<0.001
Non-Hispanic White	5,613 (67.89)	4,796 (68.26)	817 (64.71)	
Mexican American	1930 (8.38)	1720 (8.57)	210 (6.67)	
Non-Hispanic Black	2,458 (11.24)	2036 (10.58)	422 (17.01)	
Other Hispanic	1,231 (5.55)	1,055 (5.52)	176 (5.85)	
Other Race	920 (6.94)	834 (7.07)	86 (5.77)	
Smoking status, %				<0.0001
Never smoker	6,648 (56.56)	5,953 (58.64)	695 (38.33)	
Former smoker	3,035 (24.26)	2,481 (23.40)	554 (31.81)	
Current smoker	2,469 (19.18)	2007 (17.96)	462 (29.86)	
Drinking status, %				<0.0001
Non-drinker	1,629 (10.33)	1,354 (10.10)	275 (12.27)	
Former drinker	1799 (11.19)	1,343 (9.85)	456 (22.84)	
Current drinker	8,724 (78.49)	7,744 (80.04)	980 (64.89)	
BMI, kg/m^2^	29.17 (0.13)	28.77 (0.13)	32.61 (0.31)	<0.0001
BMI group, %				<0.0001
<25	3,273 (29.05)	2,993 (30.69)	280 (14.75)	
25–30	4,067 (32.12)	3,589 (32.73)	478 (26.84)	
>30	4,812 (38.82)	3,859 (36.58)	953 (58.40)	
WC	99.14 (0.34)	98.14 (0.34)	108.33 (0.75)	<0.0001
eGFR	94.91 (0.50)	96.30 (0.55)	82.69 (0.97)	<0.0001
Total energy intakes, kcal/day	2098.47 (14.49)	2127.57 (14.33)	1844.23 (31.23)	<0.0001
Total energy group, %				<0.0001
Q1	4,055 (29.55)	3,284 (28.05)	771 (42.62)	
Q2	4,131 (34.51)	3,568 (34.70)	563 (32.83)	
Q3	3,966 (35.95)	3,589 (37.25)	377 (24.55)	
Supplement use, %				<0.0001
No	7,286 (57.89)	6,359 (58.67)	927 (51.13)	
Yes	4,866 (42.11)	4,082 (41.33)	784 (48.87)	
Stroke, %				<0.0001
No	11,631 (96.80)	10,216 (98.51)	1,415 (83.06)	
Yes	503 (3.06)	216 (1.49)	287 (16.94)	
DM, %				<0.0001
No	9,755 (85.33)	8,842 (89.60)	913 (58.36)	
Yes	2,262 (13.44)	1,477 (10.40)	785 (41.64)	
Hyperlipidemia, %				<0.0001
No	3,467 (30.78)	3,174 (32.36)	293 (16.99)	
Yes	8,684 (69.22)	7,267 (67.64)	1,417 (83.01)	
Hypertension, %				<0.0001
No	6,929 (63.66)	6,524 (68.06)	405 (25.33)	
Yes	5,222 (36.33)	3,916 (31.94)	1,306 (74.67)	
Depression, %				<0.0001
No	10,961 (91.44)	9,935 (95.63)	1,026 (58.41)	
Yes	1,115 (8.10)	471 (4.37)	644 (41.59)	

BMI, body mass index; WC, waist circumference; eGFR, estimated glomerular filtration rate; DM, diabetes mellitus. Categorical variables are presented as numbers (percentages). Sampling weights were applied for calculation of demographic descriptive statistics. N reflect the study sample while percentages reflect the survey-weighted data.

To quantify the magnitude of differences between the frail and non-frail groups, standardized mean differences (SMDs) were calculated for all variables. The SMDs, which provide a standardized measure of group differences, indicated meaningful disparities (SMD ≥ 0.1) across all examined characteristics. For example, age (SMD = 0.729), body mass index (BMI, SMD = 0.518), and chronic conditions such as diabetes (SMD = 0.762) exhibited large differences between the two groups. These results underscore the importance of adjusting for these variables in subsequent analyses to mitigate potential confounding. Detailed SMD values for all variables are provided in Supplementary Table S2.

### 3.2 The association between dietary flavonoid intake and frailty

Multiple logistic regression analysis of the link between dietary flavonoid intake and the prevalence of frailty was shown in Table 2. When dietary flavonoid intake levels are analyzed as a continuous variable, most models do not show a significant association between flavonoid intake and the prevalence of frailty. Only flavanones in Model 2 (OR [95% CI] 1.00 [0.99–1.00]) suggested a potential association at the borderline significance level. When dietary flavonoid intake levels are analyzed as a categorical variable, we found that higher intakes of total flavonoids and its components were significantly linked with a lower prevalence of frailty in Model 1. Specifically, the dietary intake of flavonoids was found to be inversely associated with prevalence of frailty adjusting for Model 3. In comparison to Group 1 (reference), the following OR and 95% CIs depict these associations: Total flavonoids: Group 2 (OR = 0.88[0.74–1.06]). Group 3 (OR = 0.79[0.65–0.95]), with a p trend = 0.016; anthocyanidins: Group 2 (OR = 0.99[0.81–1.21]). Group 3 (OR = 0.71[0.58–0.88]), with a p trend = 0.002; flavanones: Group 2 (OR = 0.81[0.66–0.99]). Group 3 (OR = 0.74[0.59–0.92]), with a p trend = 0.007; flavones: Group 2 (OR = 0.84[0.72–0.98]). Group 3 (OR = 0.76[0.59–0.97]), with a p trend = 0.024; and flavonols: Group 2 (OR = 0.87[0.70–1.09]). Group 3 (OR = 0.67[0.56–0.81]), with a p trend <0.001. These results collectively underscore the robust and consistent negative associations between dietary flavonoid intake and frailty.

**Table 2:** ORs (95% CIs) of the prevalence of frailty according to dietary flavonoid intake levels (mg/day) among adults in NHANES 2007–2010 and 2017–2018.

Flavonoid type and model	Continuous flavonoid intakes		Category of flavonoid intakes			
	OR (95% CI)	*p* value	Group 1	Group 2	Group 3	*P*_trend_
Isoflavone						
Model 1	0.99 (0.97,1.00)	0.07	Ref (1.00)	0.96 (0.70,1.31)	**0.66 (0.54,0.82)**	<0.001
Model 2	0.99 (0.98,1.00)	0.12	Ref (1.00)	0.89 (0.65,1.22)	**0.73 (0.58,0.91)**	0.008
Model 3	1.00 (0.99,1.01)	0.5	Ref (1.00)	1.06 (0.76,1.47)	0.85 (0.67,1.07)	0.208
Anthocyanidins						
Model 1	1.00 (0.99,1.00)	0.33	Ref (1.00)	0.97 (0.81,1.17)	**0.67 (0.56,0.80)**	<0.0001
Model 2	1.00 (0.99,1.00)	0.14	Ref (1.00)	**0.80 (0.66,0.98)**	**0.51 (0.42,0.62)**	<0.0001
Model 3	1.00 (0.99,1.00)	0.71	Ref (1.00)	0.99 (0.81,1.21)	**0.71 (0.58,0.88)**	0.002
Flavan-3-ols						
Model 1	1.00 (1.00,1.00)	0.35	Ref (1.00)	**0.79 (0.66,0.95)**	**0.73 (0.61,0.87)**	<0.001
Model 2	1.00 (1.00,1.00)	0.23	Ref (1.00)	**0.73 (0.61,0.88)**	**0.65 (0.54,0.77)**	<0.0001
Model 3	1.00 (1.00,1.00)	0.32	Ref (1.00)	0.92 (0.75,1.14)	0.86 (0.69,1.05)	0.138
Flavanones						
Model 1	1.00 (1.00,1.00)	0.1	Ref (1.00)	**0.74 (0.62,0.89)**	**0.70 (0.58,0.84)**	<0.001
Model 2	1.00 (0.99,1.00)	0.03	Ref (1.00)	**0.67 (0.55,0.81)**	**0.56 (0.45,0.68)**	<0.0001
Model 3	1.00 (0.99,1.00)	0.28	Ref (1.00)	**0.81 (0.66,0.99)**	**0.74 (0.59,0.92)**	0.007
Flavones						
Model 1	0.96 (0.88,1.05)	0.36	Ref (1.00)	**0.81 (0.71,0.94)**	**0.63 (0.51,0.78)**	<0.0001
Model 2	0.96 (0.88,1.05)	0.41	Ref (1.00)	**0.72 (0.62,0.83)**	**0.60 (0.48,0.74)**	<0.0001
Model 3	0.99 (0.95,1.04)	0.71	Ref (1.00)	**0.84 (0.72,0.98)**	**0.76 (0.59,0.97)**	0.024
Flavonols						
Model 1	1.00 (0.99,1.00)	0.43	Ref (1.00)	**0.75 (0.63,0.91)**	**0.54 (0.45,0.66)**	<0.0001
Model 2	1.00 (0.99,1.01)	0.81	Ref (1.00)	**0.75 (0.62,0.92)**	**0.56 (0.46,0.69)**	<0.0001
Model 3	1.00 (1.00,1.01)	0.69	Ref (1.00)	0.87 (0.70,1.09)	**0.67 (0.56,0.81)**	<0.001
Total flavonoids						
Model 1	1.00 (1.00,1.00)	0.52	Ref (1.00)	**0.77 (0.65,0.91)**	**0.68 (0.57,0.81)**	<0.0001
Model 2	1.00 (1.00,1.00)	0.42	Ref (1.00)	**0.70 (0.58,0.84)**	**0.59 (0.49,0.71)**	<0.0001
Model 3	1.00 (1.00,1.00)	0.38	Ref (1.00)	0.88 (0.74,1.06)	**0.79 (0.65,0.95)**	0.016

OR, odds ratio; CI, confidence interval.

Model 1: unadjusted.

Model 2: adjusted for age (<40, 40–59, or >59 years), sex (female or male), ethnicity (Non-Hispanic White, Mexican American, Non-Hispanic Black, Other Hispanic or Other race).

Model 3: adjusted for all the factors in Model 2 and smoking status (non-smoker, former smoker, or current smoker), drinking status (non-drinker, former drinker, or current drinker), BMI (<25, 25–30, or >30), total energy intakes (in quartiles), and supplement use (yes or no).Statistical significance is indicated in bold.

**Figure 2:**
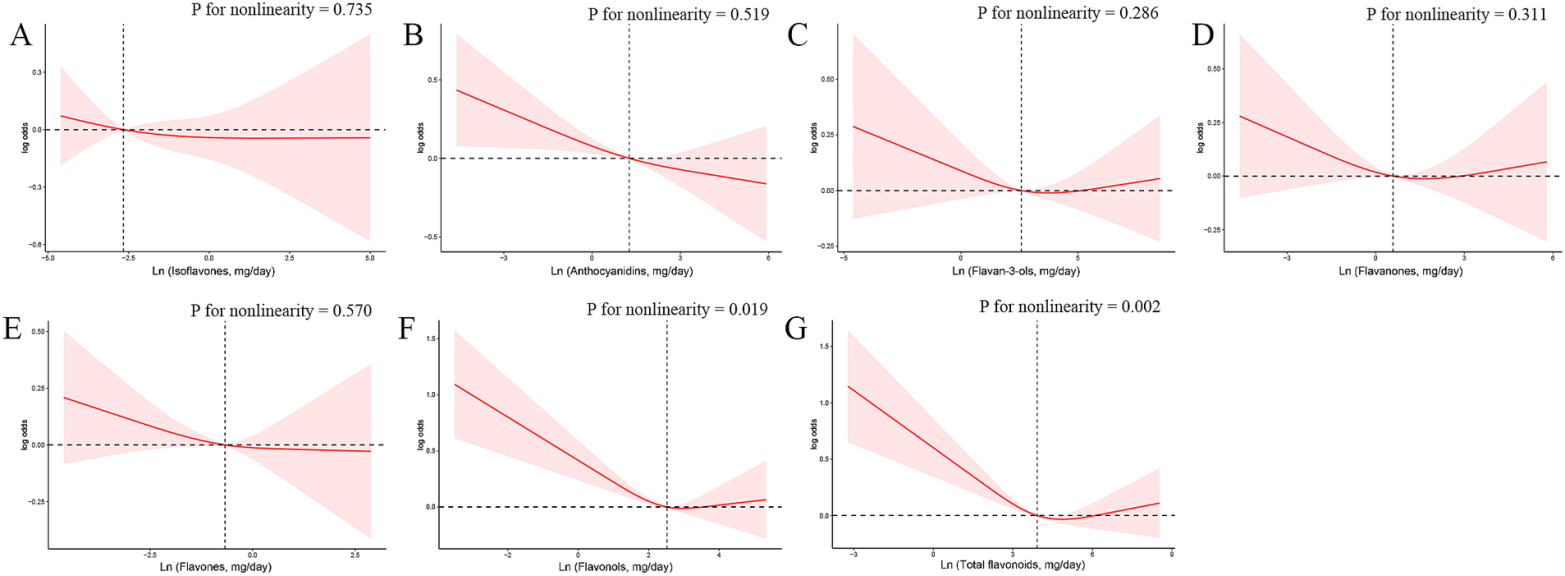
The exposure–response associations of dietary flavonoid intake [**(A)** Isoflavones; **(B)** Anthocyanidins; **(C)** Flavan-3-ols; **(D)** Flavanones; **(E)** Flavones; **(F)** Flavonols; **(G)** Total flavonoids] and frailty by restricted cubic spline (RCS) model in adults. Model was adjusted for age (<40, 40–59, or >59 years), sex (female or male), ethnicity (Non-Hispanic White, Mexican American, Non-Hispanic Black, Other Hispanic or Other race), smoking status (non-smoker, former smoker, or current smoker), drinking status (non-drinker, former drinker, or current drinker), BMI (<25, 25–30, or >30), total energy intakes (in quartiles), and supplement use (yes or no).

Subsequently, we conducted RCS analyses to explore potential nonlinear connections between the six dietary flavonoid intake categories and frailty (Figure 2). We identified a linear inverse relationship between anthocyanidins and frailty (p for overall = 0.006, p for nonlinearity = 0.519). In contrast, non-linear associations were observed between total flavonoids (p for nonlinearity = 0.002) and flavonols (p for nonlinearity = 0.019) with frailty. The discrepancy between continuous and categorical analyses may be attributed to the potential nonlinear nature of the associations, which are better captured by categorical analyses and restricted cubic spline models. The continuous analysis assumes a linear relationship, which may not adequately represent the true dose–response pattern, particularly when threshold effects exist.

The nonlinear associations between flavonols/total flavonoids and frailty were further characterized using threshold effect analysis. For flavonols, a threshold effect was observed at approximately 19.7 mg/day, with a significant inverse association below this level (OR = 0.982[0.958–1.007], *p* = 0.002) and a non-significant association above it (OR = 1.000[0.973–1.027], *p* = 0.178) (Supplementary Table S3). Similarly, for total flavonoids, a threshold effect was observed at approximately 130 mg/day, with a significant inverse association below this level (OR = 0.989[0.981–0.996], *p* = 0.006) and a non-significant association above it (OR = 0.995[0.981–1.009], *p* = 0.331). These findings suggest that moderate intake of flavonoids and flavonols may provide optimal benefits, while higher intake levels may not confer additional protective effects (Supplementary Table S4).

### 3.3 Stratified analysis

Detailed stratified analyses delineate the complex associations between dietary flavonoid intake and frailty prevalence. Supplementary Table S5 investigates age-based variation (<40, 40–59, or > 59 years) in frailty prevalence relative to flavonoid consumption. Supplementary Table S6 details gender-specific differences (female versus male) in flavonoid intake’s impact on frailty prevalence. Supplementary Table S7 examines racial disparities (Non-Hispanic White, Mexican American, Non-Hispanic Black, Other Hispanic, or Other Race – Including Multi-Racial), elucidating the interplay between dietary habits and frailty prevalence in different racial groups. Supplementary Tables S8, S9 explores the associations between flavonoid intake and frailty prevalence in different smoking and drinking status participants, respectively. The results of our stratified analyses showed interaction effects between dietary flavonoid intake and factors such as sex (Isoflavones: p for interaction = 0.043; Flavonols: p for interaction = 0.044) and drinking status (Isoflavones: p for interaction = 0.035; Total flavonoids: p for interaction = 0.026) are statistically significant, while no statistically significant interaction effects were observed in age, ethnicity, and smoking status.

To address potential concerns about collinearity in our 49-item frailty index, we conducted sensitivity analyses using the Fried phenotype definition of frailty. These analyses yielded similar patterns of association between flavonoid intake and frailty (Supplementary Table S10), supporting the robustness of our findings across different frailty definitions. To address potential residual confounding, we conducted additional sensitivity analyses adjusting for education level and Healthy Eating Index-2015. These analyses (Supplementary Table S11) showed similar patterns of association, suggesting our findings are robust to additional adjustment for these potential confounders.

### 3.4 The associations between the mixture of six dietary flavonoid intake and frailty

**Figure 3:**
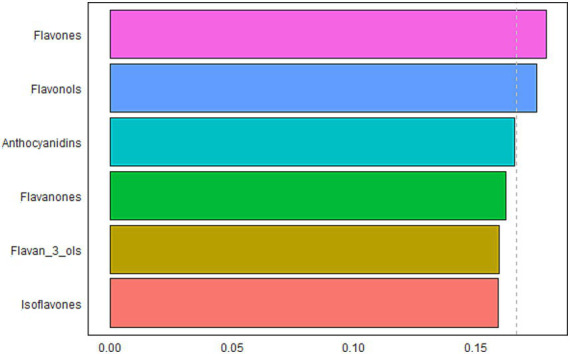
WQS model regression index weights for dietary flavonoid intakes on frailty.

The results of the WQS model to evaluate the relationship between the mixture effects of six flavonoid subclasses and frailty and identify the predominant contributors were presented in Figure 3. The WQS model demonstrated their mixed effect was inverse associated with the odds of frailty (OR: 0.58, 95% CI: 0.48–0.71, *p* < 0.001). Considering their individual effect sizes, flavones (17.9%), flavonols (17.5%), and anthocyanidins (16.6%) were the top 3 components, followed by flavanones (16.2%), flavan-3-ols (15.9%), and isoflavones (15.9%).

**Figure 4:**
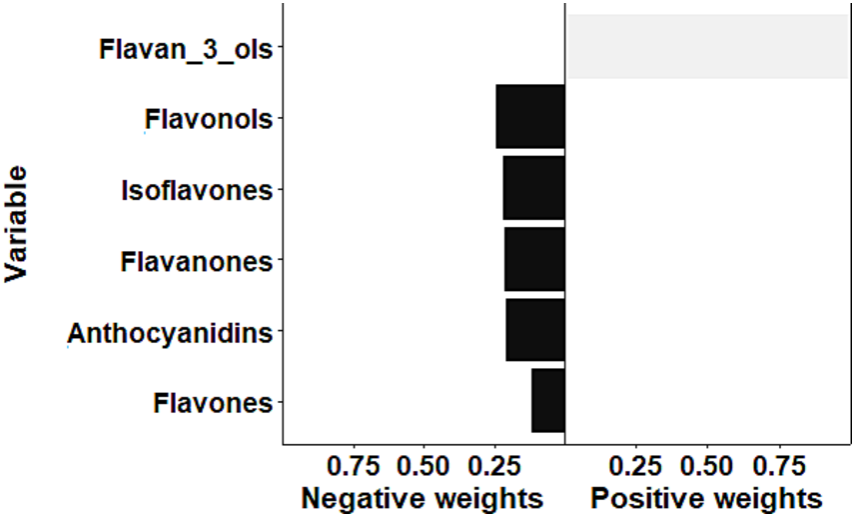
Quantile g-computation scaled weights for each flavonoid in the flavonoid mixture.

The result of sensitivity analysis by the qgcomp is illustrated in Figure 4. Overall, the qgcomp model demonstrated that the mixture of flavonoids was inversely associated with the odds of frailty (OR: 0.42, 95% CI: 0.35–0.51, *p* < 0.001), which was consistent with the above findings of the WQS model. However, both positive and negative weights were also detected via the qgcomp method. The negative weights came from flavonols (24.4%), isoflavones (21.8%), flavanones (21.3%), anthocyanidins (20.6%), and flavones (11.9%), while flavan-3-ols contributed to the positive effect. In the qgcomp model, negative weights indicate beneficial effects (reducing frailty risk), with larger absolute values suggesting stronger protective effects. The qgcomp analysis revealed that while most flavonoid subclasses showed protective associations, flavan-3-ols demonstrated potentially opposing effects. This finding may reflect complex interactions between flavonoid subclasses or different biological mechanisms at varying intake levels. Specifically, the negative weights for flavonols (24.4%), isoflavones (21.8%), and other subclasses suggest predominant protective effects, while the positive weight for flavan-3-ols indicates possible competing mechanisms.

## 4 Discussion

This comprehensive analysis provides compelling evidence of an inverse association between dietary flavonoid intake and frailty prevalence in a nationally representative sample of U.S. adults. Rather than merely confirming statistical associations, these findings have significant clinical and public health implications. The observed reduction in frailty odds with higher intakes of total flavonoids and specific subclasses (anthocyanidins, flavanones, flavones, and flavonols) suggests that dietary strategies emphasizing flavonoid-rich foods could serve as effective approaches for preventing or delaying frailty in aging populations. These protective associations remained robust after accounting for numerous potential confounding factors, reinforcing the potential causal nature of these relationships.

Our findings align with previous research suggesting beneficial effects of flavonoids on age-related health outcomes. For instance, a prospective cohort study by Samieri et al. found that higher intake of flavonoids at midlife, specifically flavones, flavanones, anthocyanins, and flavonols, is associated with a greater likelihood of health and wellbeing in individuals surviving to older ages (44). Welch et al. reported that higher flavonoid intake was associated with better bone mineral density and lower risk of osteoporosis in women (45). Similarly, Yannakoulia et al. observed that adherence to a Mediterranean diet, which is rich in flavonoids, was associated with a lower risk of frailty in older adults (46). However, Oei et al. reported no significant association between total flavonoid intake and frailty onset in the Framingham Heart Study over 12 years of follow-up, which differs from our findings (34). These contrasting results may be explained by differences in study design (cross-sectional vs. prospective), dietary assessment methods (24-h recalls vs. FFQ), and population characteristics. Meanwhile, both studies consistently found protective associations for flavonols, suggesting that specific flavonoid subclasses may be more important for frailty prevention than total flavonoid intake. These contrasting findings highlight the complexity of flavonoid-health relationships and the importance of considering methodological differences when interpreting results across studies.

The observed non-linear inverse associations for total flavonoids and flavonols are particularly intriguing. The threshold effect analysis identified inflection points at 19.7 mg/day for flavonols and 130 mg/day for total flavonoids, below which intake significantly reduced frailty risk but not above these thresholds. These findings suggest that the protective effects of flavonols and total flavonoids on frailty risk are most pronounced at lower intake levels, with diminishing returns beyond the identified thresholds. Similar non-linear relationships have been observed in other nutritional epidemiology studies. For example, Zhong et al. found a non-linear association between total flavonoid intake and the risk of NAFLD progression (47). Similarly, non-linear relationships between total flavonoid intake, flavanones, and anthocyanidins and metabolic syndrome risk have also been observed (48). The results from our mixture analysis using the Weighted Quantile Sum (WQS) regression model provide valuable insights into the relative contributions of different flavonoid subclasses. The identification of flavones, flavonols, and anthocyanidins as the top contributors to the inverse association with frailty aligns with previous research on the health benefits of these specific subclasses. For instance, Jacques et al. found that higher intake of flavonols was associated with a reduced risk of type 2 diabetes (49). Similarly, a meta-analysis by Kimble et al. reported inverse associations between anthocyanidin intake and both coronary heart disease risk and cardiovascular disease mortality (50). The sensitivity analysis using the quantile g-computation (qgcomp) model confirmed the overall inverse association between the mixture of flavonoids and frailty odds. However, the detection of both positive and negative weights for different subclasses highlights the complexity of flavonoid-health relationships and underscores the importance of considering the entire flavonoid profile rather than individual compounds in isolation. The inconsistency between WQS and qgcomp results, particularly the opposing effects of flavan-3-ols, may be attributed to the directional homogeneity assumption in WQS, which assumes all exposures have the same direction of effect. In contrast, qgcomp allows for both positive and negative weights, providing a more nuanced interpretation of the mixture effects. These models complement each other by highlighting the complexity of flavonoid-health relationships and underscoring the importance of considering the entire flavonoid profile rather than individual compounds in isolation.

The observed associations between flavonoid intake and lower frailty prevalence can be explained by several potential biological mechanisms. Flavonoids are known for their potent antioxidant and anti-inflammatory properties, which may play a crucial role in preventing or mitigating the underlying processes involved in frailty development.

Oxidative stress and inflammation are primary drivers of frailty pathophysiology. Frailty is characterized by increased oxidative stress and decreased antioxidant defenses (51). Flavonoids, particularly flavonols and anthocyanidins, possess strong antioxidant activities by scavenging free radicals, enhancing endogenous antioxidant systems, and protecting cellular membranes (52, 53). Chronic low-grade inflammation (“inflammaging”) is another hallmark of frailty (54). Flavonoids exhibit anti-inflammatory properties through inhibition of pro-inflammatory cytokine production and modulation of inflammatory signaling pathways (24). For example, quercetin, a common flavonols, has been shown to inhibit NF-κB activation and reduce the expression of inflammatory mediators (28). Flavan-3-ols primarily affect inflammatory pathways via modulation of NF-κB signaling, reduction of pro-inflammatory cytokine production, and regulation of immune cell function (21, 55). These antioxidant and anti-inflammatory effects contribute to preserving physiological reserve and resilience, key factors in frailty prevention.

Mitochondrial dysfunction increasingly appears central to frailty development and age-related decline (56). Flavonoids act as mitochondrial function modifiers, preventing damage and subsequent cellular dysfunction (57). They enhance mitochondrial biogenesis through activation of the AMPK and SIRT1 pathways, promoting PGC-1α expression (58, 59). Flavones (luteolin, apigenin) specifically maintain membrane potential, regulate fusion/fission dynamics, and enhance respiratory chain efficiency (24, 57). These improvements in mitochondrial function help maintain cellular energy production and metabolic homeostasis, potentially slowing progression toward frailty.

Flavonoids also support neural and vascular function, both crucial for frailty prevention. Cognitive decline and frailty frequently co-occur (60), and flavonoids demonstrate neuroprotective properties in both laboratory and clinical studies (61). Flavonols activate the Nrf2/ARE pathway, reduce neuronal oxidative stress, and protect against neuroinflammation (28, 61), potentially preventing cognitive frailty. Additionally, flavonoids improve endothelial function and reduce arterial stiffness (62, 63), enhancing blood flow and nutrient delivery to tissues, thereby supporting overall physiological resilience and reducing frailty risk.

Our findings are generally consistent with previous research on the relationship between flavonoid intake and age-related health outcomes. However, this study has several strengths that contribute to its novelty and significance. Firstly, while previous studies have examined the association between flavonoid intake and individual components of frailty or related conditions, our study is one of the few to investigate the relationship with a comprehensive frailty index in a large, nationally representative sample. This approach provides a more holistic assessment of frailty status and allows for a better understanding of the potential impact of flavonoids on overall physiological resilience. Secondly, the comprehensive assessment of dietary flavonoid intake using two 24-h dietary recalls and a validated flavonoid database allows for a more accurate estimation of habitual intake compared to single dietary assessments or food frequency questionnaires used in some previous studies. Thirdly, our analysis of the mixture effects of flavonoid subclasses using advanced statistical methods (WQS and qgcomp) offers new insights into the relative contributions of different flavonoids to the observed associations. This approach addresses the limitations of previous studies that often focused on individual flavonoid subclasses or total flavonoid intake without considering potential interactions or cumulative effects.

Despite these strengths, several limitations should be considered when interpreting our results. The cross-sectional design precludes causal inference, and reverse causality remains a possibility. Frail individuals may alter their dietary habits, potentially reducing their intake of flavonoid-rich foods due to functional limitations or changes in appetite. Future research directions should include prospective cohort studies to establish the temporal relationship between flavonoid intake and frailty development. Randomized controlled trials are needed to evaluate the efficacy of flavonoid-rich dietary interventions in preventing or mitigating frailty. Future studies could also investigate how flavonoids might interact with medical treatments, similar to the synergistic effects observed when combining natural compounds like Thymoquinone with chemotherapeutic agents. For instance, recent research has demonstrated enhanced therapeutic potential when 5-Fluorouracil is combined with calcium carbonate nanoparticles loaded with antioxidant Thymoquinone for cancer treatment (64, 65). While these studies focused on cancer therapeutics, similar synergistic approaches could be explored for frailty interventions, potentially combining flavonoid-rich dietary protocols with existing pharmacological or exercise-based treatments. Additionally, research into the molecular mechanisms underlying these interactions could provide further insights into the role of dietary bioactives in healthy aging and frailty prevention. While two 24-h recalls provide better estimates than single recalls, they may not fully capture long-term dietary patterns or seasonal variations in flavonoid intake. The USDA FNDDS database does account for some variations in flavonoid content, but cannot capture all sources of variability including growing conditions, processing methods, and storage. This potential misclassification would likely bias results toward the null, suggesting our findings may underestimate true associations. The assessment of frailty using a frailty index, while comprehensive, may not capture all aspects of this complex syndrome. Different frailty definitions or assessment tools might yield slightly different results. Additionally, the cross-sectional nature of the study means that reverse causality cannot be ruled out entirely; it is possible that frail individuals may have altered their dietary habits as a result of their condition. Finally, while our study population was large and diverse, certain subgroup analyses may have been limited by sample size, particularly for less common flavonoid subclasses or in specific demographic groups. This limitation may have affected our ability to detect associations in some stratified analyses. Although our study had a large and diverse sample, the significant disparity in sample sizes between non-frail and frail groups (10,441:1,711) and the predominance of non-Hispanic White participants (67.89%) may have limited the precision of certain subgroup analyses, particularly when examining less common flavonoid subclasses or specific demographic groups. Future studies should include larger and more diverse populations to validate these findings and explore potential ethnic/cultural variations in the flavonoid-frailty relationship. Additionally, mechanistic studies should explore the specific pathways through which different flavonoid subclasses influence frailty-related physiological processes. Research in diverse populations is also warranted to ensure the generalizability of our findings and to identify potential ethnic or cultural variations in the flavonoid-frailty relationship.

Our findings have important implications for public health and clinical practice. The inverse associations between flavonoid intake and frailty prevalence suggest that dietary recommendations could be modified to emphasize the consumption of flavonoid-rich foods for frailty prevention. As highlighted in a recent editorial, understanding the complex interplay between lifestyle, nutrition, and cultural factors is essential for developing effective strategies for the prevention and management of age-related conditions such as frailty (66). Public health campaigns could focus on promoting Mediterranean-style dietary patterns as suggested by Alkhatib et al. (67), which naturally incorporate diverse sources of flavonoids through increased consumption of fruits, vegetables, nuts, and olive oil. Such dietary modifications represent a feasible, cost-effective approach to reduce frailty risk at the population level. In clinical practice, healthcare providers should consider assessing and encouraging adequate flavonoid intake as part of a comprehensive approach to frailty prevention and management in older adults.

## 5 Conclusion

In conclusion, this comprehensive analysis of NHANES data provides evidence for an inverse association between dietary flavonoid intake and the prevalence of frailty in U.S. adults. Higher intakes of total flavonoids and specific subclasses, particularly flavones, flavonols, and anthocyanidins, were associated with lower odds of frailty. However, our findings cannot establish causal relationships between flavonoid intake and frailty. Longitudinal cohort studies and randomized controlled trials in the future were needed to validate our findings and establish causal relationships. These findings suggest that flavonoid-rich diets may be a promising strategy for frailty prevention and healthy aging. By elucidating the role of dietary factors in aging and frailty, our study contributes valuable insights that may inform future public health policies and dietary guidelines aimed at promoting healthy aging and reducing the burden of frailty in aging populations.

## Data Availability

Publicly available datasets were analyzed in this study. This data can be found here: The National Health and Nutrition Examination Survey dataset is publicly available at the National Center for Health Statistics of the Center for Disease Control and Prevention (https://www.cdc.gov/nchs/nhanes/index.htm).

## Footnotes

^1^ https://www.ars.usda.gov/northeast-area/beltsville-md-bhnrc/beltsville-human-nutrition-research-center/food-surveys-research-group/docs/fndds-flavonoid-database/
