# Supplementary material for: Inverse associations between dietary flavonoid and subclass intakes and frailty in U.S. adults
Source: Front Nutr. 2025 May 16;12:1490998. doi: 10.3389/fnut.2025.1490998 (PMC12122313; doi:10.3389/fnut.2025.1490998)
Supplement: Supplementary file 1 [file Data_Sheet_1.pdf]

科研诚信承诺书

题目：Inverse Associations Between Dietary Flavonoid and Subclass Intakes and Frailty in U.S. Adults

作者签名：[illegible]

编号	学术规范
1	遵循科研伦理准则，切实保障受试者权益。
2	遵守法律法规，已妥善处理研究所涉及生物安全、国家/工作秘密、个人隐私、知情同意等重大问题。
3	研究过程及结果已做到诚实记录，不存在篡改、捏造，相关研究资料已完整、准确、真实地提交所在机构统一数据库。
4	未交由“第三方”全包代做研究，代写、代投或实质性修改论文；未参与虚假审稿。
5	遵从学术规范，实事求是地陈述本人工作，按要求正确引用他人工作，不存在剽窃、抄袭或捏造。
6	论文署名的每一位作者对署名均知情，对论文有实质性贡献，并按贡献大小依序署名，不存在无贡献挂名及成果侵占。
7	成果发表时未一稿多投。
8	已做到如实全名标注资助项目，主动诚实地进行利益披露。
9	成果推广、科普宣传中秉持科学精神、坚守社会责任，未人为夸大研究基础和学术价值。

个人承诺：如违背上述承诺，本人愿接受相关部门做出的各项处理决定，包括但不限于追回资助及奖励经费，取消一定期限科研项目、人才项目、职称晋升申请资格，记入科研失信行为档案以及接受相应的党纪、政纪处理等。

承诺人：[illegible]

2024年 8月 16日
