# Supplementary material for: Inverse associations between dietary flavonoid and subclass intakes and frailty in U.S. adults
Source: Front Nutr. 2025 May 16;12:1490998. doi: 10.3389/fnut.2025.1490998 (PMC12122313; doi:10.3389/fnut.2025.1490998)
Supplement: Supplementary file 2 [file Data_Sheet_2.pdf]

论文投稿备案

打印日期:2024-09-06

基本信息

主题	论文投稿备案		
模板名称	科教/科研/KJ_论文投稿审批流程		
申请人	蔡双明	申请单编号	KJTG20240823298
部门	MICU番禺医生组	创建时间	2024-08-23 14:59
实施反馈人			

审批内容

**题目**	Inverse Associations Between Dietary Flavonoid and Subclass Intakes and Frailty in U.S. Adults
**论文初稿（包含论文名称、所有作者、通讯作者、单位、摘要，需注明通讯作者）**	论文初稿.docx
**科研诚信承诺书（所有作者按文章作者实际顺序亲笔签名）**	2024-8-16-MICU-蔡双明-Inverse Associations Between Dietary Flavonoid and Subclass Intakes and Frailty in U.S. Adults.pdf
**其他文件附件**	

论文投稿须知：

①所有论文投稿前需签署《科研诚信承诺书》，**承诺书为通讯作者负责制**，签署一式一份，第一作者本人保留，并扫描签署后的承诺函，**命名：年月-科室-第一作者或通讯名称-论文名称**，投稿前提交"论文投稿审批流程"，审批通过后论文投稿介绍信方可盖章 **（盖科教科的章即可）**

②论文相关资料和数据应当确保齐全、完整、真实和准确，所有论文在见刊后一个月内需将所涉及的原始图片、实验记录、实验数据、生物信息、记录等原始数据资料（电子版）交科教科统一管理、留存备查，**文件命名为：见刊年月-第一作者或通讯作者姓名-科室-论文名称**。提交科教科保存后科教科将开具提交原始数据证明（附件3），请**自行下载填写后到科教科盖章。**

③ **非投稿前或**无签署《科研诚信承诺书》及上交论文相关资料和数据的论文 **不予报销及奖励。**

文件模板

- 1.科研诚信承诺书.docx
- 2.已提交论文原始数据证明.docx
- 3.论文投稿介绍信.doc
- 4.《关于印发医学科研诚信和相关行为规范的通知》（国卫科教发〔2021〕7号）.pdf

1、我院标准名称有："广东省妇幼保健院、广东省儿童医院、广东省妇产医院"我院标准名称："广东省妇幼保健院"，有需要时，可以选择署名为"广东省妇幼保健院、广东省儿童医院"或"广东省妇幼保健院、广东省妇产医院"。医院英文统一名称为："Guangdong Women and Children Hospital"。
我院广医在读研究生发表文章，以"广州医科大学"为第一单位的，应该署名为："广州医科大学"，英文名称为："Guangzhou Medical University"；以"广东省妇幼保健院"为第一单位的，应按如下要求规范署名，标准署名："广东省妇幼保健院"，有需要时，可以选择署名为"广东省妇幼保健院、广东省儿童医院"或"广东省妇幼保健院、广东省妇产医院"。医院英文统一名称为："Guangdong Women and Children Hospital"。
所有文章投稿前均需走科教论文投稿审批流程，科技论文、成果等报销及奖励办法参照我院现行规定。单位名称表述不规范者将不给予相应的报销和奖励。
链接1

2、有关杂志类别，请查看《关于更新学术期刊目录的通知》
链接1

3、发票单位必须为"广东省妇幼保健院"，其他名称开具的发票无法报销。
转发中国科协办公厅等部门关于印发《发表学术论文"五不准"》的通知
链接2

审批记录

时间	节点名称	操作者	操作	处理意见
2024-08-23 15:09	起草节点	蔡双明	提交文档	
2024-08-26 08:41	科教科办理节点	李翠柳	沟通："罗毅平"	承诺书为通讯作者负责制，请通讯作者确定《科研诚信承诺书》所有作者签名是否真实、有效?
2024-08-28 08:58	科教科办理节点	罗毅平	回复："李翠柳"	真实，有效。
2024-08-28 15:42	科教科办理节点	李翠柳	审批	同意
2024-08-30 11:46	科教科审批节点	武丽	审批	同意 （来自：微信）

2024-08-30 11:46	结束节点	系统	结束流程	
