# Supplementary material for: Inverse associations between dietary flavonoid and subclass intakes and frailty in U.S. adults
Source: Front Nutr. 2025 May 16;12:1490998. doi: 10.3389/fnut.2025.1490998 (PMC12122313; doi:10.3389/fnut.2025.1490998)
Supplement: Supplementary file 3 [file Table_1.docx]

**Inverse Associations Between Dietary Flavonoid and Subclass Intakes and Frailty in U.S. Adults**

**Table S1.** Variables in the 49-Item frailty index and their respective scorings

| Variant |  |  |  |  |  | Scoring |  |  |  |  |  |  |  |  |  |  |
| --- | --- | --- | --- | --- | --- | --- | --- | --- | --- | --- | --- | --- | --- | --- | --- | --- |
| Cognition | |  |  |  |  |  |  |  |  |  |  |  |  |  |  |  |
| 1. Experience confusion/memory problems | | | | |  | Yes = 1, No = 0 | |  |  |  |  |  |  |  |  |  |
| Dependence | |  |  |  |  |  |  |  |  |  |  |  |  |  |  |  |
| 2. Managing money | | |  |  |  | Difficulty = 1, No Difficulty = 0 | | | |  |  |  |  |  |  |  |
| 3. Stooping, crouching, kneeling | | | |  |  | Difficulty = 1, No Difficulty = 0 | | | |  |  |  |  |  |  |  |
| 4. Lifting or carrying | | |  |  |  | Difficulty = 1, No Difficulty = 0 | | | |  |  |  |  |  |  |  |
| 5. House chore | |  |  |  |  | Difficulty = 1, No Difficulty = 0 | | | |  |  |  |  |  |  |  |
| 6. Preparing meals | | |  |  |  | Difficulty = 1, No Difficulty = 0 | | | |  |  |  |  |  |  |  |
| 7. Standing up from armless chair | | | |  |  | Difficulty = 1, No Difficulty = 0 | | | |  |  |  |  |  |  |  |
| 8. Getting in and out of bed difficulty | | | | |  | Difficulty = 1, No Difficulty = 0 | | | |  |  |  |  |  |  |  |
| 9. Using fork, knife, drinking from cup | | | | |  | Difficulty = 1, No Difficulty = 0 | | | |  |  |  |  |  |  |  |
| 10. Dressing yourself | | |  |  |  | Difficulty = 1, No Difficulty = 0 | | | |  |  |  |  |  |  |  |
| 11. Standing for long periods difficulty | | | | |  | Difficulty = 1, No Difficulty = 0 | | | |  |  |  |  |  |  |  |
| 12. Grasp/holding small objects | | | |  |  | Difficulty = 1, No Difficulty = 0 | | | |  |  |  |  |  |  |  |
| 13. Attending social event | | | |  |  | Difficulty = 1, No Difficulty = 0 | | | |  |  |  |  |  |  |  |
| 14. Push or pull large objects | | | |  |  | Difficulty = 1, No Difficulty = 0 | | | |  |  |  |  |  |  |  |
| 15. Walking for a quarter mile difficulty | | | | |  | Difficulty = 1, No Difficulty = 0 | | | |  |  |  |  |  |  |  |
| 16. Walking up 10 steps difficulty | | | | |  | Difficulty = 1, No Difficulty = 0 | | | |  |  |  |  |  |  |  |
| Depressive Symptoms | | |  |  |  |  |  |  |  |  |  |  |  |  |  |  |
| 17. Have little interest in doing things | | | | |  | Nearly every day = 1, More than half the days = 0.66, Several days = 0.33, Not at all = 0 | | | | | | | | | | |
| 18. Feeling down, depressed, or hopeless | | | | |  | Nearly every day = 1, More than half the days = 0.66, Several days = 0.33, Not at all = 0 | | | | | | | | | | |
| 19. Trouble sleeping or sleeping too much | | | | |  | Nearly every day = 1, More than half the days = 0.66, Several days = 0.33, Not at all = 0 | | | | | | | | | | |
| 20. Feeling tired or having little energy | | | | |  | Nearly every day = 1, More than half the days = 0.66, Several days = 0.33, Not at all =O | | | | | | | | | | |
| 21. Poor appetite or overeating | | | |  |  | Nearly every day = 1, More than half the days = 0.66, Several days = 0.33, Not at all = 0 | | | | | | | | | | |
| 22. Feeling bad about yourself | | | |  |  | Nearly every day = 1, More than half the days = 0.66, Several days = 0.33, Not at all = O | | | | | | | | | | |
| 23. Trouble concentrating on things | | | | |  | Nearly every day = 1, More than half the days = 0.66, Several days = 0.33, Not at all = 0 | | | | | | | | | | |
| Comorbidities | |  |  |  |  |  |  |  |  |  |  |  |  |  |  |  |
| 24. Arthritis | |  |  |  |  | Yes = 1, Suspect = 0.5 No = 0 | | | |  |  |  |  |  |  |  |
| 25. Thyroid problems | | |  |  |  | Yes = 1, Suspect = 0.5 No = 0 | | | |  |  |  |  |  |  |  |
| 26. Chronic bronchitis | | |  |  |  | Yes = 1, Suspect = 0.5 No = 0 | | | |  |  |  |  |  |  |  |
| 27. Cancer | |  |  |  |  | Yes = 1, Suspect = 0.5 No = 0 | | | |  |  |  |  |  |  |  |
| 28. Congestive heart failure | | | |  |  | Yes = 1, Suspect = 0.5 No = 0 | | | |  |  |  |  |  |  |  |
| 29. Coronary heart disease | | | |  |  | Yes = 1, Suspect = 0.5 No = 0 | | | |  |  |  |  |  |  |  |
| 30. Angina | |  |  |  |  | Yes = 1, Suspect = 0.5 No = 0 | | | |  |  |  |  |  |  |  |
| 31. Heart attack | |  |  |  |  | Yes = 1, Suspect = 0.5 No = 0 | | | |  |  |  |  |  |  |  |
| 32. Stroke | |  |  |  |  | Yes = 1, Suspect = 0.5 No = 0 | | | |  |  |  |  |  |  |  |
| 33. Blood pressure | | |  |  |  | Yes = 1, Suspect = 0.5 No = 0 | | | |  |  |  |  |  |  |  |
| 34. Diabetes | |  |  |  |  | Yes = 1, Suspect = 0.5 No = 0 | | | |  |  |  |  |  |  |  |
| 35. weak/failing kidneys | | |  |  |  | Yes = 1, Suspect = 0.5 No = 0 | | | |  |  |  |  |  |  |  |
| 36. Urinary Leakage | | |  |  |  | Yes = 1, Suspect = 0.5 No = 0 | | | |  |  |  |  |  |  |  |
| Hospital Utilization and Access to Care | | | | |  |  |  |  |  |  |  |  |  |  |  |  |
| 37. Self-rated health | | |  |  |  | Fair, poor = 1, Excellent, Very good, good = 0 | | | | | |  |  |  |  |  |
| 38. Health now compared with 1 year ago | | | | |  | Worse = 1, About the same, better = 0 | | | | |  |  |  |  |  |  |
| 39. Overnight hospital patient in past year | | | | | | Yes = 1, No = 0 | |  |  |  |  |  |  |  |  |  |
| 40. Frequency of health care use during past year | | | | | | None = 0, 1-5 = 0,5, More than 5 = 1 | | | | |  |  |  |  |  |  |
| 41. Number of prescribed medications | | | | |  | None = 0, 1-4 = 0.5, 5 and more = 1 | | | | |  |  |  |  |  |  |
| Physical Performance and Anthropometry | | | | |  |  |  |  |  |  |  |  |  |  |  |  |
| 42. Body mass index | | |  |  |  | <18.5, >30 = 1 | |  |  |  |  |  |  |  |  |  |
|  |  |  |  |  |  | 25-<30 = 0.5 | |  |  |  |  |  |  |  |  |  |
|  |  |  |  |  |  | 18.5-25 = 0 | |  |  |  |  |  |  |  |  |  |
| 43. Handgrip strength | | |  |  |  | MALE: |  |  |  | FEMALE: |  |  |  |  |  |  |
|  |  |  |  |  |  | For BMI ≤ 24, GS ≤ 29 | | |  | For BMI ≤ 23, GS ≤ 17 | | |  |  |  |  |
|  |  |  |  |  |  | For BMI 24.1-28, GS ≤ 30 | | |  | For BMI 23.1-26, GS ≤ 17.3 | | | |  |  |  |
|  |  |  |  |  |  | For BMI >28, GS ≤ 32 = 1 | | |  | For BMI 26.1-29, GS ≤ 18 | | |  |  |  |  |
|  |  |  |  |  |  |  |  |  |  | For BMI>29, GS ≤ 21 = 1 | | |  |  |  |  |
| Laboratory Values | | |  |  |  |  |  |  |  |  |  |  |  |  |  |  |
| 44. Glycohemoglobin (%) | | |  |  |  | 0%—5.7% = 0, >5.7% = 1 | | |  |  |  |  |  |  |  |  |
| 45. Red blood cell count (million cells/uL) | | | | | | M: 4.7-6.1 = 0, Other = 1 | | |  | F: 4.2-5.4 = 0, Other = 1 | | |  |  |  |  |
| 46. Hemoglobin (g/dL.) | | |  |  |  | M: 13.5-18 = 0, Other = 1 | | |  | F: 12-16 = 0, Other = 1 | | |  |  |  |  |
| 47. Red cell distribution width (%) | | | | |  | 11.6-14.6 = 0, Other = 1 | | |  |  |  |  |  |  |  |  |
| 48. Lymphocyte percent (%) | | | |  |  | 20-40 = 0, Other = 1 | | |  |  |  |  |  |  |  |  |
| 49. Segmented neutrophils percent (%) | | | | |  | 40-80 = 0, Other = 1 | | |  |  |  |  |  |  |  |  |
| BMI, Body mass index; GS, grip strength. | | | | |  |  |  |  |  |  |  |  |  |  |  |  |

**Table S2.** Standardized Mean Differences (SMDs) Between Frail and Non-Frail Groups

| Characteristics | Non-frailty  (n = 10,441) | Frailty  (n = 1,711) | SMD |
| --- | --- | --- | --- |
| Age,years | 45.72(0.35) | 57.19(0.55) | 0.729 |
| Sex,% |  |  | 0.236 |
| Female | 5287(51.22) | 1023(62.84) |  |
| Male | 5154(48.78) | 688(37.16) |  |
| Race/ethnicity,% |  |  | 0.200 |
| Non-Hispanic White | 4796(68.26) | 817(64.71) |  |
| Mexican American | 1720(8.57) | 210(6.67) |  |
| Non-Hispanic Black | 2036(10.58) | 422(17.01) |  |
| Other Hispanic | 1055(5.52) | 176(5.85) |  |
| Other Race | 834(7.07) | 86(5.77) |  |
| Smoking status,% |  |  | 0.421 |
| Never smoker | 5953(58.64) | 695(38.33) |  |
| Former smoker | 2481(23.40) | 554(31.81) |  |
| Current smoker | 2007(17.96) | 462(29.86) |  |
| Drinking status,% |  |  | 0.380 |
| Non-drinker | 1354(10.10) | 275(12.27) |  |
| Former drinker | 1343( 9.85) | 456(22.84) |  |
| Current drinker | 7744(80.04) | 980(64.89) |  |
| BMI,kg/m^2^ | 28.77(0.13) | 32.61(0.31) | 0.518 |
| WC | 98.14(0.34) | 108.33(0.75) | 0.600 |
| eGFR | 96.30(0.55) | 82.69(0.97) | 0.581 |
| Total energy intakes, kcal/day | 2127.57(14.33) | 1844.23(31.23) | 0.349 |
| Supplement use, % |  |  | 0.152 |
| No | 6359(58.67) | 927(51.13) |  |
| Yes | 4082(41.33) | 784(48.87) |  |
| Stroke,% |  |  | 0.555 |
| No | 10216(98.51) | 1415(83.06) |  |
| Yes | 216( 1.49) | 287(16.94) |  |
| DM,% |  |  | 0.762 |
| No | 8842(89.60) | 913(58.36) |  |
| Yes | 1477(10.40) | 785(41.64) |  |
| Hyperlipidemia,% |  |  | 0.362 |
| No | 3174(32.36) | 293(16.99) |  |
| Yes | 7267(67.64) | 1417(83.01) |  |
| Hypertension,% |  |  | 0.948 |
| No | 6524(68.06) | 405(25.33) |  |
| Yes | 3916(31.94) | 1306(74.67) |  |
| Depression,% |  |  | 0.987 |
| No | 9935(95.63) | 1026(58.41) |  |
| Yes | 471( 4.37) | 644(41.59) |  |

BMI, body mass index; WC, waist circumference; eGFR, estimated glomerular filtration rate; DM, diabetes mellitus. Categorical variables are presented as numbers (percentages). Sampling weights were applied for calculation of demographic descriptive statistics. N reflect the study sample while percentages reflect the survey-weighted data.

**Table S3.** Threshold effect analysis of flavonols on frailty risk

|  | Adjusted OR (95%CI) | P value |
| --- | --- | --- |
| Fitting by standard linear model | 0.990(0.984,0.996) | 0.004 |
| Fitting by two-piecewise linear model |  |  |
| Inflection point |  |  |
| ≤ 19.7 mg/day | 0.982(0.958,1.007) | 0.002 |
| ＞19.7 mg/day | 1.000(0.973,1.027) | 0.178 |
| P for Log-likelihood ratio |  | 0.043 |

**Table S4.** Threshold effect analysis of total flavonoids on frailty risk

|  | Adjusted OR (95%CI) | P value |
| --- | --- | --- |
| Fitting by standard linear model | 0.995(0.991,0.999) | 0.012 |
| Fitting by two-piecewise linear model |  |  |
| Inflection point |  |  |
| ≤ 130 mg/day | 0.989(0.981,0.996) | 0.006 |
| ＞130 mg/day | 0.995(0.981,1.009) | 0.331 |
| P for Log-likelihood ratio |  | 0.069 |

**Table S5.** Stratified analyses of the prevalence of frailty according to dietary flavonoid intake levels (mg/day) by age (< 40, 40–59, or > 59 years) in NHANES 2007–2010 and 2017–2018.

| Flavonoids | <39 | 40-59 | >59 |
| --- | --- | --- | --- |
| Isoflavones |  |  |  |
| Group 1 | Ref (1.00) | Ref (1.00) | Ref (1.00) |
| Group 2 | 0.688(0.334,1.419) | 1.392(0.834,2.325) | 0.928(0.626,1.375) |
| Group 3 | 0.422(0.245,0.727) | 0.980(0.685,1.401) | 0.874(0.647,1.181) |
| p for trend | 0.002 | 0.924 | 0.362 |
| p for interaction | 0.134 |  |  |
| Anthocyanidins |  |  |  |
| Group 1 | Ref (1.00) | Ref (1.00) | Ref (1.00) |
| Group 2 | 0.837(0.507,1.381) | 1.011(0.744,1.374) | 1.068(0.778,1.466) |
| Group 3 | 0.808(0.395,1.655) | 0.543(0.370,0.797) | 0.862(0.636,1.168) |
| p for trend | 0.509 | 0.002 | 0.265 |
| p for interaction | 0.16 |  |  |
| Flavan-3-ols |  |  |  |
| Group 1 | Ref (1.00) | Ref (1.00) | Ref (1.00) |
| Group 2 | 0.597(0.380,0.939) | 0.972(0.742,1.271) | 0.969(0.659,1.424) |
| Group 3 | 0.892(0.463,1.721) | 0.886(0.638,1.230) | 0.849(0.606,1.190) |
| p for trend | 0.624 | 0.455 | 0.31 |
| p for interaction | 0.436 |  |  |
| Flavanones |  |  |  |
| Group 1 | Ref (1.00) | Ref (1.00) | Ref (1.00) |
| Group 2 | 0.767(0.475,1.238) | 0.935(0.637,1.374) | 0.718(0.525,0.982) |
| Group 3 | 0.450(0.255,0.794) | 0.800(0.568,1.125) | 0.729(0.549,0.967) |
| p for trend | 0.007 | 0.199 | 0.032 |
| p for interaction | 0.669 |  |  |
| Flavones |  |  |  |
| Group 1 | Ref (1.00) | Ref (1.00) | Ref (1.00) |
| Group 2 | 0.824(0.487,1.394) | 0.793(0.578,1.087) | 0.883(0.693,1.125) |
| Group 3 | 0.538(0.291,0.996) | 0.656(0.455,0.944) | 0.906(0.621,1.321) |
| p for trend | 0.047 | 0.023 | 0.598 |
| p for interaction | 0.26 |  |  |
| Flavonols |  |  |  |
| Group 1 | Ref (1.00) | Ref (1.00) | Ref (1.00) |
| Group 2 | 0.627(0.332,1.184) | 0.932(0.652,1.333) | 0.854(0.623,1.170) |
| Group 3 | 0.702(0.430,1.145) | 0.674(0.506,0.897) | 0.650(0.461,0.917) |
| p for trend | 0.129 | 0.007 | 0.017 |
| p for interaction | 0.716 |  |  |
| Total flavonoids |  |  |  |
| Group 1 | Ref (1.00) | Ref (1.00) | Ref (1.00) |
| Group 2 | 0.832(0.563,1.228) | 0.743(0.534,1.033) | 1.008(0.780,1.303) |
| Group 3 | 1.048(0.584,1.880) | 0.738(0.525,1.038) | 0.792(0.577,1.087) |
| p for trend | 0.918 | 0.076 | 0.124 |
| p for interaction | 0.193 |  |  |

Analyses were adjusted for covariates age (< 40, 40–59, or > 59 years), sex (female or male), ethnicity (Non-Hispanic White, Mexican American, Non-Hispanic Black, Other Hispanic or Other race), smoking status (never smoker, former smoker, or current smoker), drinking status (non-drinker, former drinker, or current drinker), BMI (<25, 25-30, or >30), total energy intakes (in quartiles), and supplement use (yes or no) when they were not the strata variables.

**Table S6.** Stratified analyses of the prevalence of frailty according to dietary flavonoid intake levels (mg/day) by sex (female or male) in NHANES 2007–2010 and 2017–2018.

| Flavonoids | Female | Male |
| --- | --- | --- |
| Isoflavones |  |  |
| Group 1 | Ref (1.00) | Ref (1.00) |
| Group 2 | 1.356(0.902,2.039) | 0.659(0.456,0.952) |
| Group 3 | 0.875(0.620,1.234) | 0.802(0.597,1.079) |
| p for trend | 0.626 | 0.107 |
| p for interaction | 0.043 |  |
| Anthocyanidins |  |  |
| Group 1 | Ref (1.00) | Ref (1.00) |
| Group 2 | 1.029(0.785,1.351) | 0.944(0.711,1.252) |
| Group 3 | 0.826(0.616,1.108) | 0.549(0.391,0.771) |
| p for trend | 0.182 | 0.001 |
| p for interaction | 0.373 |  |
| Flavan-3-ols |  |  |
| Group 1 | Ref (1.00) | Ref (1.00) |
| Group 2 | 0.890(0.663,1.194) | 0.943(0.739,1.204) |
| Group 3 | 0.993(0.740,1.332) | 0.661(0.504,0.865) |
| p for trend | 0.978 | 0.003 |
| p for interaction | 0.07 |  |
| Flavanones |  |  |
| Group 1 | Ref (1.00) | Ref (1.00) |
| Group 2 | 0.875(0.685,1.117) | 0.716(0.538,0.954) |
| Group 3 | 0.746(0.592,0.940) | 0.725(0.530,0.992) |
| p for trend | 0.016 | 0.036 |
| p for interaction | 0.375 |  |
| Flavones |  |  |
| Group 1 | Ref (1.00) | Ref (1.00) |
| Group 2 | 0.950(0.765,1.181) | 0.700(0.518,0.946) |
| Group 3 | 0.921(0.674,1.257) | 0.566(0.403,0.794) |
| p for trend | 0.584 | 0.002 |
| p for interaction | 0.21 |  |
| Flavonols |  |  |
| Group 1 | Ref (1.00) | Ref (1.00) |
| Group 2 | 1.035(0.781,1.372) | 0.631(0.471,0.844) |
| Group 3 | 0.832(0.651,1.064) | 0.480(0.353,0.653) |
| p for trend | 0.161 | <0.0001 |
| p for interaction | 0.044 |  |
| Total flavonoids |  |  |
| Group 1 | Ref (1.00) | Ref (1.00) |
| Group 2 | 1.042(0.808,1.342) | 0.686(0.511,0.921) |
| Group 3 | 0.961(0.755,1.223) | 0.572(0.405,0.806) |
| p for trend | 0.729 | 0.002 |
| p for interaction | 0.16 |  |

Analyses were adjusted for covariates age (< 40, 40–59, or > 59 years), sex (female or male), ethnicity (Non-Hispanic White, Mexican American, Non-Hispanic Black, Other Hispanic or Other race), smoking status (never smoker, former smoker, or current smoker), drinking status (non-drinker, former drinker, or current drinker), BMI (<25, 25-30, or >30), total energy intakes (in quartiles), and supplement use (yes or no) when they were not the strata variables.

**Table S7.** Stratified analyses of the prevalence of frailty according to dietary flavonoid intake levels (mg/day) by ethnicity (Non-Hispanic White, Mexican American, Non-Hispanic Black, Other Hispanic or Other race) in NHANES 2007–2010 and 2017–2018.

| Flavonoids | Non-Hispanic White | Mexican American | Non-Hispanic Black | Other Hispanic | Other Race |
| --- | --- | --- | --- | --- | --- |
| Isoflavones |  |  |  |  |  |
| Group 1 | Ref (1.00) | Ref (1.00) | Ref (1.00) | Ref (1.00) | Ref (1.00) |
| Group 2 | 1.130(0.766,1.666) | 0.981(0.500,1.926) | 0.530(0.329,0.853) | 0.916(0.307,2.738) | 2.286(0.799,6.542) |
| Group 3 | 0.878(0.650,1.186) | 0.875(0.560,1.366) | 0.771(0.525,1.132) | 0.760(0.438,1.319) | 0.708(0.376,1.334) |
| p for trend | 0.491 | 0.546 | 0.094 | 0.341 | 0.358 |
| p for interaction | 0.232 |  |  |  |  |
| Anthocyanidins |  |  |  |  |  |
| Group 1 | Ref (1.00) | Ref (1.00) | Ref (1.00) | Ref (1.00) | Ref (1.00) |
| Group 2 | 0.970(0.733,1.285) | 0.774(0.460,1.305) | 1.074(0.750,1.539) | 1.381(0.640,2.981) | 0.983(0.447,2.161) |
| Group 3 | 0.687(0.512,0.922) | 0.789(0.394,1.578) | 0.890(0.624,1.271) | 1.097(0.566,2.129) | 0.395(0.159,0.982) |
| p for trend | 0.012 | 0.494 | 0.578 | 0.816 | 0.029 |
| p for interaction | 0.606 |  |  |  |  |
| Flavan-3-ols |  |  |  |  |  |
| Group 1 | Ref (1.00) | Ref (1.00) | Ref (1.00) | Ref (1.00) | Ref (1.00) |
| Group 2 | 0.916(0.680,1.234) | 0.931(0.532,1.630) | 0.929(0.666,1.295) | 0.894(0.518,1.544) | 1.201(0.478,3.019) |
| Group 3 | 0.888(0.657,1.200) | 0.607(0.297,1.243) | 0.935(0.674,1.297) | 0.750(0.356,1.581) | 0.772(0.343,1.740) |
| p for trend | 0.431 | 0.192 | 0.67 | 0.437 | 0.435 |
| p for interaction | 0.977 |  |  |  |  |
| Flavanones |  |  |  |  |  |
| Group 1 | Ref (1.00) | Ref (1.00) | Ref (1.00) | Ref (1.00) | Ref (1.00) |
| Group 2 | 0.799(0.614,1.039) | 0.438(0.251,0.764) | 0.936(0.601,1.459) | 1.171(0.597,2.296) | 0.850(0.328,2.204) |
| Group 3 | 0.677(0.515,0.890) | 0.658(0.343,1.263) | 0.860(0.576,1.283) | 0.678(0.342,1.344) | 1.437(0.652,3.165) |
| p for trend | 0.006 | 0.199 | 0.457 | 0.261 | 0.377 |
| p for interaction | 0.201 |  |  |  |  |
| Flavones |  |  |  |  |  |
| Group 1 | Ref (1.00) | Ref (1.00) | Ref (1.00) | Ref (1.00) | Ref (1.00) |
| Group 2 | 0.886(0.714,1.098) | 0.696(0.401,1.208) | 0.798(0.554,1.151) | 0.830(0.455,1.515) | 0.462(0.207,1.033) |
| Group 3 | 0.837(0.623,1.125) | 0.577(0.299,1.113) | 0.788(0.549,1.132) | 0.474(0.237,0.949) | 0.443(0.176,1.116) |
| p for trend | 0.224 | 0.1 | 0.155 | 0.033 | 0.122 |
| p for interaction | 0.865 |  |  |  |  |
| Flavonols |  |  |  |  |  |
| Group 1 | Ref (1.00) | Ref (1.00) | Ref (1.00) | Ref (1.00) | Ref (1.00) |
| Group 2 | 0.905(0.678,1.207) | 0.946(0.459,1.950) | 0.819(0.587,1.142) | 0.476(0.257,0.882) | 0.821(0.427,1.581) |
| Group 3 | 0.712(0.550,0.921) | 0.639(0.269,1.521) | 0.839(0.590,1.191) | 0.421(0.194,0.915) | 0.333(0.141,0.787) |
| p for trend | 0.011 | 0.337 | 0.276 | 0.021 | 0.009 |
| p for interaction | 0.211 |  |  |  |  |
| Total flavonoids |  |  |  |  |  |
| Group 1 | Ref (1.00) | Ref (1.00) | Ref (1.00) | Ref (1.00) | Ref (1.00) |
| Group 2 | 0.936(0.734,1.193) | 0.881(0.484,1.605) | 0.893(0.593,1.346) | 0.650(0.339,1.250) | 0.616(0.305,1.245) |
| Group 3 | 0.824(0.640,1.060) | 0.693(0.333,1.445) | 0.880(0.586,1.319) | 0.604(0.258,1.416) | 0.549(0.267,1.128) |
| p for trend | 0.124 | 0.33 | 0.516 | 0.23 | 0.154 |
| p for interaction | 0.966 |  |  |  |  |

Analyses were adjusted for covariates age (< 40, 40–59, or > 59 years), sex (female or male), ethnicity (Non-Hispanic White, Mexican American, Non-Hispanic Black, Other Hispanic or Other race), smoking status (never smoker, former smoker, or current smoker), drinking status (non-drinker, former drinker, or current drinker), BMI (<25, 25-30, or >30), total energy intakes (in quartiles), and supplement use (yes or no) when they were not the strata variables.

**Table S8.** Stratified analyses of the prevalence of frailty according to dietary flavonoid intake levels (mg/day) by smoking status (Never smoker, Former smoker or Current smoker) in NHANES 2007–2010 and 2017–2018.

| Flavonoids | Never smoker | Former smoker | Current smoker |
| --- | --- | --- | --- |
| Isoflavones |  |  |  |
| Group 1 | Ref (1.00) | Ref (1.00) | Ref (1.00) |
| Group 2 | 1.081(0.700,1.668) | 1.053(0.605,1.834) | 0.965(0.536,1.738) |
| Group 3 | 0.855(0.601,1.217) | 0.877(0.598,1.286) | 0.777(0.540,1.118) |
| p for trend | 0.407 | 0.543 | 0.19 |
| p for interaction | 0.968 |  |  |
| Anthocyanidins |  |  |  |
| Group 1 | Ref (1.00) | Ref (1.00) | Ref (1.00) |
| Group 2 | 1.109(0.801,1.536) | 0.904(0.633,1.291) | 1.028(0.759,1.393) |
| Group 3 | 0.805(0.602,1.077) | 0.761(0.504,1.148) | 0.547(0.359,0.834) |
| p for trend | 0.096 | 0.184 | 0.012 |
| p for interaction | 0.441 |  |  |
| Flavan-3-ols |  |  |  |
| Group 1 | Ref (1.00) | Ref (1.00) | Ref (1.00) |
| Group 2 | 0.936(0.676,1.298) | 0.959(0.677,1.361) | 0.881(0.597,1.300) |
| Group 3 | 0.901(0.643,1.263) | 0.904(0.611,1.339) | 0.758(0.489,1.175) |
| p for trend | 0.547 | 0.604 | 0.211 |
| p for interaction | 0.98 |  |  |
| Flavanones |  |  |  |
| Group 1 | Ref (1.00) | Ref (1.00) | Ref (1.00) |
| Group 2 | 0.839(0.589,1.194) | 0.530(0.382,0.736) | 1.152(0.763,1.739) |
| Group 3 | 0.763(0.538,1.083) | 0.664(0.445,0.990) | 0.757(0.486,1.181) |
| p for trend | 0.13 | 0.038 | 0.329 |
| p for interaction | 0.045 |  |  |
| Flavones |  |  |  |
| Group 1 | Ref (1.00) | Ref (1.00) | Ref (1.00) |
| Group 2 | 0.860(0.651,1.136) | 0.783(0.554,1.106) | 0.904(0.640,1.276) |
| Group 3 | 0.925(0.673,1.273) | 0.681(0.427,1.085) | 0.594(0.358,0.987) |
| p for trend | 0.661 | 0.103 | 0.044 |
| p for interaction | 0.264 |  |  |
| Flavonols |  |  |  |
| Group 1 | Ref (1.00) | Ref (1.00) | Ref (1.00) |
| Group 2 | 0.891(0.685,1.161) | 0.873(0.562,1.357) | 0.847(0.542,1.322) |
| Group 3 | 0.679(0.464,0.994) | 0.691(0.477,1.000) | 0.635(0.441,0.915) |
| p for trend | 0.042 | 0.057 | 0.02 |
| p for interaction | 0.998 |  |  |
| Total flavonoids |  |  |  |
| Group 1 | Ref (1.00) | Ref (1.00) | Ref (1.00) |
| Group 2 | 1.017(0.745,1.388) | 0.806(0.560,1.162) | 0.829(0.535,1.287) |
| Group 3 | 0.917(0.632,1.332) | 0.756(0.505,1.134) | 0.675(0.474,0.963) |
| p for trend | 0.622 | 0.173 | 0.031 |
| p for interaction | 0.654 |  |  |

Analyses were adjusted for covariates age (< 40, 40–59, or > 59 years), sex (female or male), ethnicity (Non-Hispanic White, Mexican American, Non-Hispanic Black, Other Hispanic or Other race), smoking status (never smoker, former smoker, or current smoker), drinking status (non-drinker, former drinker, or current drinker), BMI (<25, 25-30, or >30), total energy intakes (in quartiles), and supplement use (yes or no) when they were not the strata variables.

**Table S9.** Stratified analyses of the prevalence of frailty according to dietary flavonoid intake levels (mg/day) by drinking status (Non-drinker, Former drinker or Current drinker) in NHANES 2007–2010 and 2017–2018.

| Flavonoids | Non-drinker | Former drinker | Current drinker |
| --- | --- | --- | --- |
| Isoflavones |  |  |  |
| Group 1 | Ref (1.00) | Ref (1.00) | Ref (1.00) |
| Group 2 | 0.553(0.307,0.997) | 0.652(0.355,1.195) | 1.269(0.855,1.884) |
| Group 3 | 0.984(0.648,1.495) | 0.654(0.386,1.106) | 0.886(0.663,1.183) |
| p for trend | 0.726 | 0.088 | 0.559 |
| p for interaction | 0.035 |  |  |
| Anthocyanidins |  |  |  |
| Group 1 | Ref (1.00) | Ref (1.00) | Ref (1.00) |
| Group 2 | 1.408(0.991,2.000) | 0.871(0.635,1.194) | 0.977(0.741,1.290) |
| Group 3 | 1.180(0.731,1.906) | 0.663(0.439,0.999) | 0.666(0.510,0.869) |
| p for trend | 0.518 | 0.047 | 0.004 |
| p for interaction | 0.276 |  |  |
| Flavan-3-ols |  |  |  |
| Group 1 | Ref (1.00) | Ref (1.00) | Ref (1.00) |
| Group 2 | 1.064(0.646,1.753) | 0.868(0.606,1.245) | 0.921(0.695,1.222) |
| Group 3 | 1.012(0.590,1.736) | 0.619(0.382,1.001) | 0.913(0.671,1.243) |
| p for trend | 0.973 | 0.055 | 0.563 |
| p for interaction | 0.39 |  |  |
| Flavanones |  |  |  |
| Group 1 | Ref (1.00) | Ref (1.00) | Ref (1.00) |
| Group 2 | 0.942(0.618,1.436) | 0.737(0.512,1.061) | 0.815(0.633,1.049) |
| Group 3 | 0.959(0.603,1.525) | 0.649(0.424,0.994) | 0.728(0.563,0.942) |
| p for trend | 0.842 | 0.039 | 0.017 |
| p for interaction | 0.58 |  |  |
| Flavones |  |  |  |
| Group 1 | Ref (1.00) | Ref (1.00) | Ref (1.00) |
| Group 2 | 1.021(0.622,1.674) | 0.722(0.482,1.082) | 0.847(0.704,1.020) |
| Group 3 | 1.080(0.562,2.078) | 0.575(0.351,0.942) | 0.762(0.560,1.037) |
| p for trend | 0.815 | 0.027 | 0.08 |
| p for interaction | 0.368 |  |  |
| Flavonols |  |  |  |
| Group 1 | Ref (1.00) | Ref (1.00) | Ref (1.00) |
| Group 2 | 1.179(0.673,2.068) | 0.789(0.521,1.196) | 0.833(0.599,1.157) |
| Group 3 | 0.947(0.558,1.607) | 0.607(0.406,0.908) | 0.637(0.495,0.818) |
| p for trend | 0.922 | 0.017 | <0.001 |
| p for interaction | 0.794 |  |  |
| Total flavonoids |  |  |  |
| Group 1 | Ref (1.00) | Ref (1.00) | Ref (1.00) |
| Group 2 | 1.317(0.829,2.095) | 0.630(0.450,0.882) | 0.913(0.729,1.145) |
| Group 3 | 1.089(0.654,1.814) | 0.505(0.333,0.766) | 0.844(0.650,1.096) |
| p for trend | 0.75 | 0.002 | 0.199 |
| p for interaction | 0.026 |  |  |

Analyses were adjusted for covariates age (< 40, 40–59, or > 59 years), sex (female or male), ethnicity (Non-Hispanic White, Mexican American, Non-Hispanic Black, Other Hispanic or Other race), smoking status (never smoker, former smoker, or current smoker), drinking status (non-drinker, former drinker, or current drinker), BMI (<25, 25-30, or >30), total energy intakes (in quartiles), and supplement use (yes or no) when they were not the strata variables.

**Table S10.** Association of dietary flavonoid intake with frailty phenotype(modified) among adults in NHANES 2007–2010 and 2017–2018

|  | Category of flavonoid intakes | | | |
| --- | --- | --- | --- | --- |
|  | Group 1 | Group 2 | Group 3 | *P* _trend_ |
| Isoflavone |  |  |  |  |
| Model 1 | Ref (1.00) | 1.09(0.90,1.30) | **0.83(0.72,0.96)** | 0.023 |
| Model 2 | Ref (1.00) | 1.03(0.85,1.26) | 0.88(0.75,1.02) | 0.122 |
| Model 3 | Ref (1.00) | 1.09(0.89,1.33) | 0.94(0.81,1.09) | 0.463 |
| Anthocyanidins |  |  |  |  |
| Model 1 | Ref (1.00) | 1.22(1.03,1.45) | 0.91(0.77,1.07) | 0.223 |
| Model 2 | Ref (1.00) | 1.09(0.91,1.29) | **0.75(0.62,0.91)** | 0.003 |
| Model 3 | Ref (1.00) | 1.17(0.98,1.40) | 0.84(0.69,1.01) | 0.056 |
| Flavan-3-ols |  |  |  |  |
| Model 1 | Ref (1.00) | 1.01(0.85,1.19) | 0.99(0.85,1.15) | 0.844 |
| Model 2 | Ref (1.00) | 0.98(0.82,1.16) | 0.91(0.77,1.07) | 0.220 |
| Model 3 | Ref (1.00) | 1.04(0.88,1.25) | 0.99(0.84,1.16) | 0.820 |
| Flavanones |  |  |  |  |
| Model 1 | Ref (1.00) | 0.85(0.72,1.01) | **0.78(0.67,0.91)** | 0.002 |
| Model 2 | Ref (1.00) | **0.78(0.64,0.93)** | **0.67(0.57,0.78)** | <0.0001 |
| Model 3 | Ref (1.00) | **0.81(0.67,0.98)** | **0.72(0.61,0.85)** | <0.001 |
| Flavones |  |  |  |  |
| Model 1 | Ref (1.00) | 0.92(0.77,1.10) | **0.73(0.61,0.87)** | <0.001 |
| Model 2 | Ref (1.00) | 0.85(0.70,1.03) | **0.69(0.57,0.83)** | <0.001 |
| Model 3 | Ref (1.00) | 0.90(0.73,1.09) | **0.74(0.61,0.89)** | 0.002 |
| Flavonols |  |  |  |  |
| Model 1 | Ref (1.00) | 0.84(0.70,1.01) | **0.75(0.62,0.90)** | 0.003 |
| Model 2 | Ref (1.00) | 0.86(0.71,1.04) | **0.77(0.63,0.94)** | 0.013 |
| Model 3 | Ref (1.00) | 0.89(0.73,1.08) | **0.80(0.65,0.98)** | 0.034 |
| Total flavonoids |  |  |  |  |
| Model 1 | Ref (1.00) | 0.96(0.82,1.11) | 0.87(0.75,1.02) | 0.091 |
| Model 2 | Ref (1.00) | 0.92(0.78,1.07) | **0.79(0.67,0.94)** | 0.009 |
| Model 3 | Ref (1.00) | 0.99(0.84,1.15) | 0.86(0.72,1.03) | 0.094 |

OR, odds ratio; CI, confidence interval.

Model 1: unadjusted;

Model 2: adjusted for age (< 40, 40–59, or > 59 years), sex (female or male), ethnicity (Non-Hispanic White, Mexican American, Non-Hispanic Black, Other Hispanic or Other race);

Model 3: adjusted for all the factors in Model 2 and smoking status (non-smoker, former smoker, or current smoker), drinking status (non-drinker, former drinker, or current drinker), BMI (<25, 25-30, or >30), total energy intakes (in quartiles), and supplement use (yes or no).

**Construction of the modified frailty phenotype**

From 2003 to 2004 on, gait speed-one of the five original criteria was no longer captured in NHANES participants over the age of 50. Barreto and colleagues validated a four-item frailty phenotype, demonstrating that it can still identify people at risk of adverse health outcomes (1). As such, we created a modified four-item phenotype using four of the Wilhelm-Leen et al. criteria (2):

- exhaustion, defined by “some difficulty”, “much difficulty”, or “unable to do” when asked how much difficulty they have “walking from one room to the other on the same level”.
- low physical activity, defined as ≤600 MET-min/week, because the question “Compared with most (men/women) your age, would you say that you are more active, less active, or about the same?” was no longer captured from 2007 to 2008 cycle on.
- weakness, defined by “some difficulty”, “much difficulty”, or “unable to do” when asked how much difficulty they have “lifting or carrying something as heavy as 10 pounds [like a sack of potatoes or rice]”.
- low body weight, defined by BMI ≤18.5 kg/m^2^.

Previous literature defined frail individuals as having 3 or 4 of the items, pre-frail individuals as having 1 or 2 of the items and robust individuals as having no items. If an individual had missing data for any of the 4 items, they were excluded from the study. In this study, we combined frailty and pre-frailty for analysis.

Reference

1. Barreto Pde S, Greig C, Ferrandez AM. Detecting and categorizing frailty status in older adults using a self-report screening instrument. Archives of gerontology and geriatrics. 2012;54(3):e249-54. doi.org/10.1016/j.archger.2011.08.003.

2. Wilhelm-Leen ER, Hall YN, M KT, Chertow GM. Frailty and chronic kidney disease: the Third National Health and Nutrition Evaluation Survey. The American journal of medicine. 2009;122(7):664-71.e2. doi.org/10.1016/j.amjmed.2009.01.026.

**Table S11.** Association of dietary flavonoid intake with frailty when adjusted for education level and Healthy Eating Index -2015.

|  | Category of flavonoid intakes | | | |
| --- | --- | --- | --- | --- |
|  | Group 1 | Group 2 | Group 3 | *P* _trend_ |
| Isoflavone |  |  |  |  |
| Model 1 | Ref (1.00) | 0.96(0.70,1.31) | **0.66(0.54,0.82)** | <0.001 |
| Model 2 | Ref (1.00) | 1.04(0.76,1.42) | **0.71(0.57,0.88)** | 0.005 |
| Model 3 | Ref (1.00) | 1.01(0.74,1.38) | **0.71(0.57,0.88)** | 0.003 |
| Anthocyanidins |  |  |  |  |
| Model 1 | Ref (1.00) | 0.97(0.81,1.17) | **0.67(0.56,0.80)** | <0.0001 |
| Model 2 | Ref (1.00) | 1.02(0.85,1.22) | **0.76(0.64,0.90)** | 0.003 |
| Model 3 | Ref (1.00) | 1.03(0.87,1.23) | **0.75(0.62,0.91)** | 0.004 |
| Flavan-3-ols |  |  |  |  |
| Model 1 | Ref (1.00) | **0.79(0.66,0.95)** | **0.73(0.61,0.87)** | <0.001 |
| Model 2 | Ref (1.00) | 0.86(0.71,1.03) | **0.83(0.69,0.99)** | 0.036 |
| Model 3 | Ref (1.00) | 0.85(0.69,1.05) | **0.78(0.64,0.96)** | 0.019 |
| Flavanones |  |  |  |  |
| Model 1 | Ref (1.00) | **0.74(0.62,0.89)** | **0.70(0.58,0.84)** | <0.001 |
| Model 2 | Ref (1.00) | **0.82(0.69,0.97)** | **0.78(0.65,0.94)** | 0.009 |
| Model 3 | Ref (1.00) | **0.77(0.65,0.93)** | **0.76(0.62,0.93)** | 0.007 |
| Flavones |  |  |  |  |
| Model 1 | Ref (1.00) | **0.81(0.71,0.94)** | **0.63(0.51,0.78)** | <0.0001 |
| Model 2 | Ref (1.00) | 0.86(0.74,1.00) | **0.72(0.58,0.90)** | 0.004 |
| Model 3 | Ref (1.00) | **0.85(0.73,0.99)** | **0.68(0.55,0.84)** | <0.001 |
| Flavonols |  |  |  |  |
| Model 1 | Ref (1.00) | **0.75(0.63,0.91)** | **0.54(0.45,0.66)** | <0.0001 |
| Model 2 | Ref (1.00) | 0.80(0.66,0.97) | 0.60(0.50,0.73) | <0.0001 |
| Model 3 | Ref (1.00) | **0.78(0.65,0.94)** | **0.58(0.48,0.69)** | <0.0001 |
| Total flavonoids |  |  |  |  |
| Model 1 | Ref (1.00) | **0.77(0.65,0.91)** | **0.68(0.57,0.81)** | <0.0001 |
| Model 2 | Ref (1.00) | **0.84(0.71,0.98)** | **0.79(0.65,0.94)** | 0.010 |
| Model 3 | Ref (1.00) | 0.84(0.70,1.00) | **0.74(0.61,0.90)** | 0.003 |

OR, odds ratio; CI, confidence interval.

Model 1: unadjusted;

Model 2: adjusted for education level;

Model 3: adjusted for Healthy Eating Index -2015.
